# Synergic Antitumor Effect of Photodynamic Therapy and Chemotherapy Mediated by Nano Drug Delivery Systems

**DOI:** 10.3390/pharmaceutics14020322

**Published:** 2022-01-29

**Authors:** Mozhgan Aghajanzadeh, Mostafa Zamani, Fereshteh Rajabi Kouchi, Josh Eixenberger, Dorsa Shirini, David Estrada, Farhad Shirini

**Affiliations:** 1 Department of Chemistry, College of Science, University of Guilan, Rasht 41335-19141, Iran; m.aghajanzadeh.k@gmail.com (M.A.); m.zamani.r1990@gmail.com (M.Z.); 2 Micron School of Materials Science and Engineering, Boise State University, Boise, ID 83725, USA; fereshtehrajabik@u.boisestate.edu (F.R.K.); daveestrada@boisestate.edu (D.E.); 3 Center for Advanced Energy Studies, Boise State University, Boise, ID 83725, USA; 4 School of Medicine, Shahid Beheshti University of Medical Sciences, Tehran 1985717443, Iran; Dorsa74@gmail.com

**Keywords:** nano-platforms, nano photosensitizers, synergistic effect, combination of photodynamic therapy/chemotherapy, drug delivery systems, cancer

## Abstract

This review provides a summary of recent progress in the development of different nano-platforms for the efficient synergistic effect between photodynamic therapy and chemotherapy. In particular, this review focuses on various methods in which photosensitizers and chemotherapeutic agents are co-delivered to the targeted tumor site. In many cases, the photosensitizers act as drug carriers, but this review, also covers different types of appropriate nanocarriers that aid in the delivery of photosensitizers to the tumor site. These nanocarriers include transition metal, silica and graphene-based materials, liposomes, dendrimers, polymers, metal–organic frameworks, nano emulsions, and biologically derived nanocarriers. Many studies have demonstrated various benefits from using these nanocarriers including enhanced water solubility, stability, longer circulation times, and higher accumulation of therapeutic agents/photosensitizers at tumor sites. This review also describes novel approaches from different research groups that utilize various targeting strategies to increase treatment efficacy through simultaneous photodynamic therapy and chemotherapy.

**Table of Contents**

1. **Introduction** ........................................................................................................................................................................3

 1.1. Principles of Photodynamic Therapy .........................................................................................................................3

 1.2. Mechanism of Photodynamic Therapy ......................................................................................................................4

 1.3. Photosensitizers ............................................................................................................................................................5

2. **Combination of Photodynamic Therapy and Chemotherapy** .....................................................................................5

 2.1. Combination of Photosensitizers and Chemo-Drugs without External Carriers..................................................5

   2.1.1. Photosensitizers as Carriers .............................................................................................................................5

     MXenes..................................................................................................................................................................6

   2.1.2. Photosensitizer-Drug Materials .......................................................................................................................9

 2.2. Combination of Photosensitizers and Chemo-Drugs with External Carriers .......................................................9

   2.2.1. Transition Metal Based Nano-Platforms .......................................................................................................12

     Synthesis Routes of Transition Metals Nano-Platforms.................................................................................12

     Application of Transition Metals in PDT.........................................................................................................13

   2.2.2. Silica ..................................................................................................................................................................14

     Synthesis Routes of Silica..................................................................................................................................15

     Application of Silica in PDT..............................................................................................................................15

   2.2.3. Graphene ..........................................................................................................................................................16

     Application of Graphene in PDT......................................................................................................................17

   2.2.4. Liposomes ........................................................................................................................................................18

     Synthesis Routes of Liposomes........................................................................................................................19

     Application of Liposome in PDT......................................................................................................................19

   2.2.5. Dendrimers ......................................................................................................................................................20

   2.2.6. Polymers ..........................................................................................................................................................20

     Application of Dendrimers in PDT..................................................................................................................20

   2.2.7. Metal-Organic Frameworks ...........................................................................................................................20

     Main Synthesizing Methods.............................................................................................................................20

     Application of Polymers in PDT......................................................................................................................20

   2.2.8. Biological Nanocarriers .................................................................................................................................24

     Preparation Method of Metal–Organic Frameworks....................................................................................24

     Application of Metal–Organic Frameworks in PDT......................................................................................24

   2.2.9. Nanoemulsions ...............................................................................................................................................25

     Preparation of Red Blood Cells Membranes-Derived Vesicles....................................................................25

     Application of Biological Nanocarriers in PDT.............................................................................................25

   2.2.10. Nano Emulsions............................................................................................................................................26

     Synthesis Routes of Nano Emulsions..............................................................................................................26

     Application of Nano Emulsion in PDT...........................................................................................................26

 2.3. Targeting Strategy......................................................................................................................................................26

   2.3.1. pH Triggered...................................................................................................................................................27

   2.3.2. Enzyme Triggered...........................................................................................................................................28

   2.3.3. Redox Triggered Agents.................................................................................................................................28

   2.3.4. Chemical and Biological Targeting Agents...................................................................................................29

3. **Conclusions and Outlook** ..............................................................................................................................................30

## 1. Introduction

In 2019, 1,762,450 new cancer cases and 606,880 cancer deaths were projected to occur in the United States alone [1]. Common treatment strategies utilized to treat various cancers include surgery, radiotherapy, and chemotherapy, which can be invasive and result in serious short- and/or long-term side effects [2]. For instance, the mechanism-of-action in many traditional chemotherapeutics interferes with cell division and is often associated with severe systemic adverse effects such as myelosuppression, mucositis, alopecia, and others. Other therapeutic options have their own drawbacks. Surgical resection of certain tumors results in a high recurrence rate. Radiation therapy can be effective, but the cumulative radiation dose puts a hard limit on radiotherapy [3,4,5]. Due to the complicated burdens that these treatments can have on patients, new protocols and technologies are needed to improve treatment options and patient outcomes [6]. Photodynamic therapy (PDT) is one of these new promising approaches [7]. PDT is a treatment that involves the delivery of a photosensitizer (PS) through topical or other systemic options and is followed by irradiating the targeted tissue with a specific wavelength of light that is tailored to the given photosensitizer [8]. Among the various types of photosensitizers, those that can absorb visible or infrared light are more favorable due to the lower toxicity of infrared in comparison with ultraviolet light. PDT can also be used before, after, or in combination with more traditional treatment strategies. One advantage of many PSs is that they do not accumulate in the nuclei of the cells, preventing them from being carcinogenic by themselves [6]. Furthermore, it significantly reduces side effects traditionally observed when compared to chemotherapy or radiotherapy [9]. However, its efficacy against metastatic cancers vs. primary tumors is still questionable as it requires light irradiation, and thus the location of any secondary tumors must be known to be effective [10].

Some studies have recently investigated the efficacy of combining chemotherapy and PDT [11]. For example, different in vitro studies have shown that the combination of photosensitizer with chemotherapeutic agents such as meso-tetrahydroxyphenylchlorine and cisplatin were more effective than either therapy alone [12,13,14]. Therefore, in this review, recent advances in the combination of photodynamic therapy and chemotherapy are covered.

### 1.1. Principles of Photodynamic Therapy

In 1903, von Tappeiner and Jesionek proposed the first published report on the use of PDT as a treatment for skin tumors by using tropical eosin and exposing it to light [15,16]. They observed that oxygen was a significant part of the events found by Raab and co-workers, and introduced the term “photodynamic action”. Research on using PDT as a treatment for different tumors continued into the 1950s and 1960s by some research groups [17]. In these reports, PDT had a great advantage compared to conventional therapies as they demonstrated that they could limit toxicity to the tumor site, therefore protecting healthy cells to reduce off target effects. Since then, PDT has been applied to the treatment of non-malignant diseases in the field of dermatology, ophthalmology, urology, immunology, etc. [18].

### 1.2. Mechanism of Photodynamic Therapy

**Figure 1:**
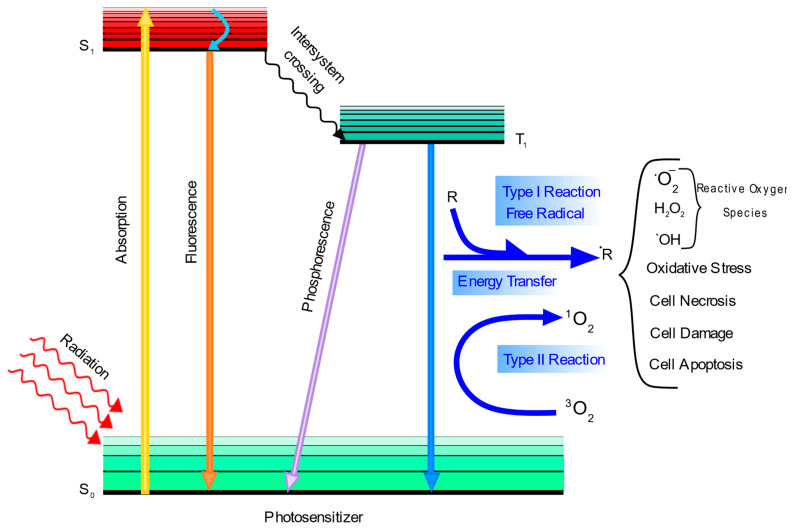
Mechanism of photodynamic therapy (figure created with Inkscape).

In PDT, a photosensitizer should be excited by a specific wavelength of light that causes two different types of reactions to occur: type I and type II photochemical reactions [19]. As shown in Figure 1, a photosensitizer’s electrons can be excited from the ground state to the excited singlet state via light, which can cause three different situations. First, the exited electron can decay back to the ground state and emit fluorescence. Second, an intersystem crossing can occur to form a triplet state that is more stable than the singlet state. This triplet state can either decay back to the ground state via emitting phosphorescence or it may interact with the environment to generate radicals.

Type I reactions occur where the triplet state forms radicals with biomolecules such as lipid radicals that can further react with other biomolecules and then oxygen to form reactive oxygen species (ROS) such as hydroxyl radicals and hydrogen peroxides. Additionally, these excited electrons can react directly with molecular oxygen to produce the superoxide anion radical, which can form other ROS species [20]. Type II reactions occur if the energy of the excited photosensitizer is transferred to a triplet oxygen in the ground state to generate a singlet oxygen. Even though both reactions (I and II) can be damaging to cells, it has been proposed that reaction type II may be more important in vivo for PDT. There are a large number of biomolecules such as proteins, lipids, and nucleic acids that react with radicals generated by PDT. This phenomenon damages biomolecules and subsequently damages tumor cells so that necrosis, apoptosis, or autophagy occurs. Additionally, immune responses against tumor cells may be activated by PDT-induced vascular injury [21,22,23]. Furthermore, chemotherapy can help PDT to be more efficient. This combination can provide long-term tumor control because of its synergistic effect on improving the efficiency of cancer treatment.

### 1.3. Photosensitizers

Although pharmaceutical companies have conducted a lot of research, finding an appropriate and effective photosensitizer is still a significant challenge, and there are only a few photosensitizers that are currently approved for the clinical treatment of cancer [24]. There are four different factors that can contribute to the efficiency of a photosensitizer: ^1^O_2_ yield (singlet oxygen), the distribution of PSs, depth penetration of the light, and molecule stability. The generation of singlet oxygen is a very important factor for PDT because of its extreme cytotoxicity in PDT [25]. Additionally, heavy atoms such as bromine and iodine can be incorporated into photosensitizers whereby interactions between triplet oxygen and native radicals can be inhibited, which increases singlet oxygen generation. One major hurdle to overcome when finding a suitable PS for therapeutic treatment is the lack of stability of many photosensitizers. This fact has led to a lot of research that aims to modify the structure of photosensitizers to increase their stability or improve their efficacy. Modification can also increase either the efficiency of converting light into singlet oxygen by adding electron donor molecules that improve the efficiency of the photosensitizers’ light absorption and/or increase the targeting ability of photosensitizers by adding ligands such as folate, peptide, and biotin [26].

## 2. Combination of Photodynamic Therapy and Chemotherapy

There are three different types of photosensitizers, which are presented in Table 1. Photosensitizers can be utilized as carriers to deliver various types of therapeutics to the targeted site. Therapeutics may be conjugated via chemical bonds or adsorbed to the surface of the PS via van der Waals forces [27]. Some photosensitizers such as the Ru(II) arene complex, [(η6-p-cymene)Ru(2,3-bis(2-pyridyl)-benzoquinoxaline)(pyridine)]^2+^, can act as a dual functional agent where these PS materials are both the photosensitizer and the chemotherapeutic [28]. Additionally, drug delivery systems that have been referred to as third generation photosensitizers have been used to improve the efficiency of photosensitizers such as enhancing the drug’s accumulation in the tumor site.

### 2.1. Combination of Photosensitizers and Chemo-Drugs without External Carriers

#### 2.1.1. Photosensitizers as Carriers

Many photosensitizers have been reported as drug carriers (Table 2). 

For example, in 2019, Cui and co-workers [49] demonstrated a semiconducting polymer containing grafted polyethylene glycol. This polymer not only showed photodynamic activity, but it was also a suitable carrier to conjugate other chemotherapeutic drugs such as bromoisophosphoramide. This work was the first reported about hypoxia-activatable phototherapeutic polymeric prodrug systems. This is a semiconducting polymer nano-prodrug (SPNpd) that can efficiently generate singlet oxygen (^1^O_2_) under near-infrared (NIR) photo-irradiation and activate its chemotherapeutic action in a hypoxic tumor microenvironment.

Lim and coworkers also [29] reported a nanocarrier that formed via host–guest interaction between oxaliplatin-adamantane prodrug and porphyrin as a photosensitizer to achieve stimulus-responsive combination therapy [50]. Oxaliplatin and porphyrin were separately modified with β-cyclodextrin and adamantane to synthesize the amphiphilic host−guest system for self-assembly into therapeutic nanoparticles. This redox-responsive system aids oxaliplatin-adamantane to be inactive until it accumulates in tumor cells. This phenomena can decrease the side effects on healthy cells.

##### MXenes

MXenes are a relatively new class of two dimensional (2D) materials that consist of transition metals (M) and carbides, nitrides, or carbonitrides (X). The chemical formula of MXenes is $M_{n+1}X_{n}T_{X}\;\left(n=1,\;2,\;\mathrm{or}\;3\right)$, where M is an early transition metal (e.g., Ti, Mo, Cr, Zr,Sc, V, Ta, Nb or Hf), X is carbon and/or nitrogen, and T_X_ stands for the terminal functional groups (e.g.,=O, OH, and F) found on the surface of MXene flakes [51,52,53,54]. The type and ratio of functional groups depends on the preparation method, post-synthesis steps, and the storage conditions of MXenes. The terminal functional groups with hydrophilic nature endow MXenes with highly hydrophilic properties. Several unique properties such as their excellent conductivity, impressive mechanical properties, and good thermal conductivity makes MXenes highly suited for various applications such as electrochemical energy storage, water purification, electrocatalysis, optoelectronics, biomedicine, and sensors [55,56,57,58].

**Figure 2:**
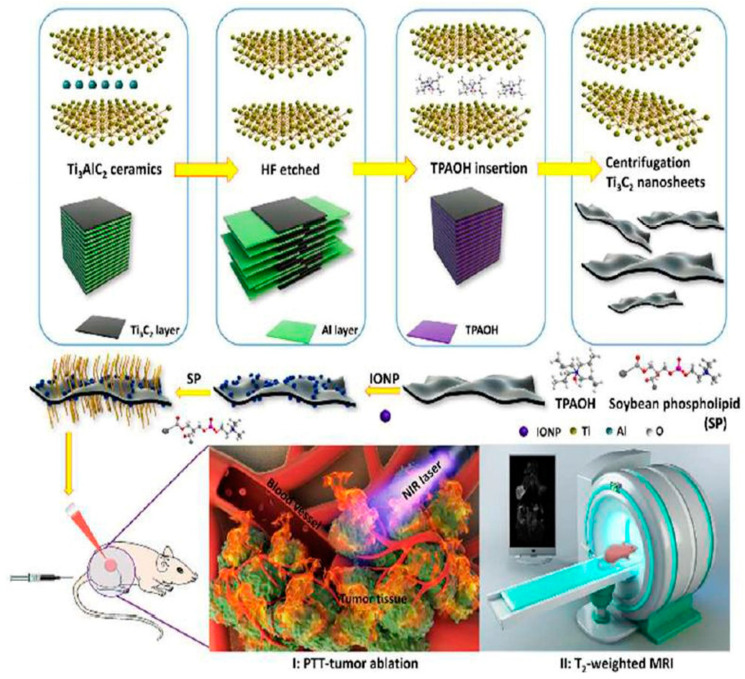
Exfoliation process and surface engineering of magnetic 2D Ti_3_C_2_-IONPs-SPs nanocomposites and their multifunctionalities for tumor theranostics. Photoacoustic imaging: PS, and iron oxide nanoparticles: IONP. Reproduced with permission from [60], The Royal Society of Chemistry, 2018.

Certain properties of MXenes make them more suitable for biomedical applications compared to other 2D nanomaterials. The hydrophilic nature of MXenes, due to the functional groups on the surface, enhances their dispersibility in biological samples. Additionally, the planar 2D structure of MXenes endows them with a very high surface-to-volume ratio, providing abundant sites for attaching various molecules such as therapeutics, targeting moieties, and other surface modification to improve biocompatibility. Dai and coworkers [59] synthesized MnO_x_/Ti_3_C_2_ composites through a simple redox reaction of KMnO_4_ (a strong oxidizing agent), which can react with OH groups on the surface of Ti_3_C_2_ nanosheets and generates paramagnetic MnO_X_ species simultaneously. Further modification with soybean phospholipids (SP) enhanced both the stability of MnO_x_/Ti_3_C_2_ composite nanosheets, but more importantly, the photo-thermal-conversion performance for killing tumor cells via PTT. Moreover, MnO_x_/Ti_3_C_2_ nanocomposites demonstrated high biocompatibility, which broadens the potential biomedical applications that surface modified MXenes could be used for. In addition, Liu and coworkers [60] (Figure 2) reported Ti_3_C_2_-IONPs MXene composites synthesized by in situ growth of superparamagnetic Fe_3_O_4_ nanocrystals on the surface of Ti_3_C_2_ MXenes. This composite exhibited a higher photo-thermal conversion efficiency than bare Ti_3_C_2_. It also showed high biocompatibility in vitro and in vivo without causing observable toxicity to cells and mice. MXenes also have strong optical absorption in both first and second NIR (NIR-I and NIR-II) biological windows [61,62], and they exhibit high photo-thermal conversion efficiency, providing potential applications of MXenes for photoacoustic imaging (PAI) and cancer phototherapy (both PDT and photo thermal therapy).

**Preparation of MXenes:** The outstanding properties and great potential applications of MXenes have led researchers to explore novel synthesis methods of new MXenes [63,64,65]. More than 100 different MXenes have been studied theoretically by computational models, and more than 30 different types have been produced and studied experimentally [66,67]. These compounds can be synthesized similar to other 2D materials via top–down and bottom–up approaches. However, the top–down approach is generally used to synthesize MXenes in order to enable large-scale production and minimize costs. MXenes are experimentally synthesized by selectively etching the “A” element from the MAX phase that has the chemical formula $M_{n+1}{A\;X}_{n}$, where M is a transition metal, A is an A-group (group 13–16) element, and X is carbon and/or nitrogen [68]. MAX phases are composed of A layers sandwiched between hexagonal transition metal carbides and nitrides. Although the strength of bond between elements M and A is weaker than between elements M and X, mechanical exfoliation cannot break the metallic bonds of M–A and requires etching away the A layers with F containing etchants such as hydrofluoric acid (HF), a mixture of hydrochloric acid/lithium fluoride (HCl/LiF), or ammonium hydrogen bifluoride (NH_4_HF_2_) [54,69,70,71]. During etching, the A layers are replaced by functional groups such as OH, O, or F. The number and the types of functional groups on the surface of MXenes heavily depend on the type of MAX phase, the type of etchant, and the synthesis method [51]. In order to separate the layers of m-MXenes to single layer MXenes, the intercalation step is necessary by introducing large organic solvents such as dimethyl sulfoxide (DMSO), tetrabutylammonium hydroxide (TBAOH), tetramethylammonium hydroxide (TMAOH), and metal cations such as Li^+^ to increase the interlayer spacing and weaken the interactions between layers [51,72,73]. After the intercalation step, sonication (bath or tip) or hand shaking can delaminate the MXene sheets from each other [54].

**Application of MXenes in PDT:** MXenes have been utilized for various anticancer therapy applications. They have been used in many different modes such as nano-platforms in drug delivery, photo-thermal agents for photo-thermal therapy, and as photosensitizers (ROS generation) for PDT [66,74]. Liu and co-workers [41] reported the reactive oxygen species (ROS) generation capabilities of Ti_3_C_2_ MXene nanosheets under irradiation. They developed a surface modification method to synthesize a small lateral size of Ti_3_C_2_ MXene nanosheets with functional groups of Al(OH)_4_^−^ by supplying additive Al^3+^ during the etching step. This new composition exhibited an excellent mass extinction coefficient (28.6 Lg^−1^cm^−1^ at 808 nm) and outstanding photo-thermal conversion (~58.3%) at 808 nm laser irradiation due to the enhanced localized surface plasmon resonance (LSPR) effect. These modifications also produced a negatively charged surface on the Ti_3_C_2_ nanosheets. They used this feature to utilize the layer-by-layer adsorption method to load doxorubicin on the surface of the Ti_3_C_2_ nanosheets and hyaluronic acid as the active tumor targeting agent. Additionally, this modification enhanced the synergistic PDT/photo thermal therapy/chemotherapy by killing cancer cells in both in vitro and in vivo experiments [41]. In another study, Bai and co-workers [42] synthesized a multifunctional Ti_3_C_2_ MXene (Ti_3_C_2_@Met@CP) via layer-by-layer adsorption of metformin (Met) as a antitumor drug and compound polysaccharide (CP) on the surface of the Ti_3_C_2_ nanosheet, which resulted in a high loading capacity of Met and CP. After the preparation of (AlOH)_4_^−^ functionalized ultrathin Ti_3_C_2_ nanosheets, Met was loaded on the surface of Ti_3_C_2_ as a chemotherapy drug to increase the adjuvant treatment. Then, to improve biocompatibility and endow and activate immune function, CP was loaded on the surface of the Ti_3_C_2_@Met composite nanosheet. A mixture of lentinan, pachymaran, and tremella polysaccharide in an optimal ratio was mixed and called CP, showing better anticancer and activating immune function effects than a single polysaccharide. The CP shell also effectively prevents the release of Met in the process of blood circulation. In vivo and in vitro experiments demonstrated that Ti_3_C_2_ composite nanosheets have excellent stability, which enhanced the effective ablation of tumors. In parallel, the photodynamic behavior of Ti_3_C_2_ composite nanosheets was investigated using DPBF and $2^{\prime },7^{\prime }$-dicholofluorscein diacetate (DCFH-DA) as a detector for in vivo and in vitro, respectively, showing the potential of Ti_3_C_2_ composite nanosheets as a new PS and to generate ROS upon 808 nm irradiation for PDT. Therefore, both in vitro and in vivo experiments have shown that Ti_3_C_2_@Met@CP composite nanosheets not only have an excellent synergistic therapeutic effect of PDT, photo-thermal therapy, and chemotherapy, but also have the ability to activate the immune system. This feature caused the complete eradication of the tumor and inhibited tumor recurrence and metastasis. The mechanism of ^1^O_2_ formation in Ti_3_C_2_ (Ti_3_C_2_@Met@CP and Ti_3_C_2_-DOX) involves the transfer of the energy of photo-excited electrons from Ti_3_C_2_ to ^3^O_2_. The exact mechanism is currently unknown with most published papers focusing on the application of MXenes in photo thermal therapy, of which there are currently only a few.

#### 2.1.2. Photosensitizer-Drug Materials

**Figure 3:**
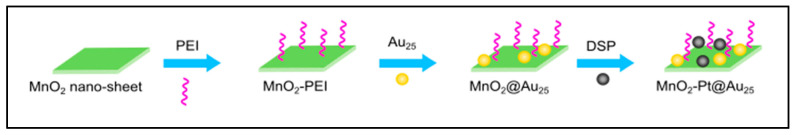
MnO_2_-Pt@Au_25_ nano-platform: a system that can be considered as both drugs and photosensitizer. Polyethylene imine: PEI (figure created with Inkscape).

Chen and co-workers [28] developed the Ru(II) arene complex, [(η6-p-cymene)Ru(2,3-bis(2-pyridyl)-benzoquinoxaline)(pyridine)]^2+^, which is able to generate ^1^O_2_, and its ligand can be dissociated under irradiation with visible light to demonstrate dual potential for PDT and photoactivated chemotherapy (Table 2). The distorted coordination geometry of 2,3-bis(2-pyridyl)-benzoquinoxaline, which is due to its bulky nature, assists Ru to be more exposable to fragment nucleic bases of DNA. In 2018, Yang and co-workers [36] introduced a novel biodegradable photosensitizer formulated as MnO_2_-Pt@Au_25_ for dual PDT and chemotherapy. In this nano-platform, manganese oxide can react with glutathione, whereby it can improve the efficiency of PDT (Figure 3).

### 2.2. Combination of Photosensitizers and Chemo-Drugs with External Carriers

**Figure 4:**
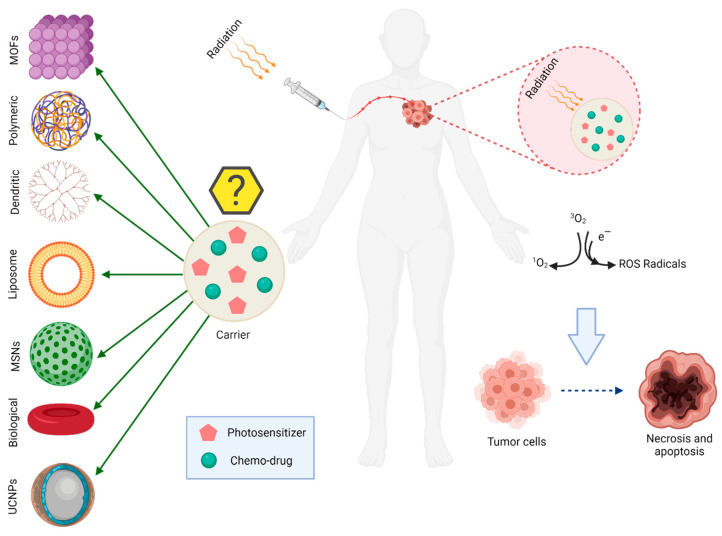
Different carriers have been used for the co-delivery of photosensitizers and drugs (figure created with BioRender.com, access 17 January 2021).

Different strategies have been used to improve the efficiency of photosensitizers such as the addition of receptor ligands and/or using nanocarriers to actively improve drug delivery efficiency and enhance drug accumulation in the tumor site. These drug delivery systems have been referred to as third generation photosensitizers and much research has been conducted in this area recently. Therefore, it is expected that the development of different types of photosensitizers will enhance their efficiency and will gain more significant applications in clinical treatments (Table 3 and Figure 4) [18].

#### 2.2.1. Transition Metal Based Nano-Platforms

The most important materials that are used as both nanocarriers and photosensitizers are prepared based on transition metals. Photosensitizer systems based on transition metals have been significantly used due to their ability to be triggered by near infrared light, which is less harmful than ultraviolet light for the human body and has a greater tissue penetration depth. On the other hand, most nano-platforms have up-conversion ability, which is the ability of emitting visible or ultraviolet light by absorbing near infrared [33]. Different transition metals such as Cr, Mn, Fe, Co, Ni, Cu, and Zn can be combined or doped to make nano-platform systems that can decrease the energy gap of transition metals to the range of visible and infrared light. Iron can combine with oxygen and a third metal to form one of the well-known metal nano-platforms. Spinel ferrites, which are shown by the MFe_2_O_4_ (M = the third metal) formula, usually with magnetic properties, are among new types of hybrid materials that can be suitable platforms for several other applications such as drug carriers and photosensitizers.

##### Synthesis Routes of Transition Metals Nano-Platforms

There are several methods for synthesizing transition metal nano-platforms. Among them, hydrothermal, co-precipitation, and micro emulsion are the most common. Most of these methods are convenient, environmentally friendly, and inexpensive. In the hydrothermal method, metal salts are dissolved in water and heated for 24 h at about 100 °C in an oven. Then, the dried sample is milled to form a powder. Finally, calcination is used to crystallize the product [151]. In the coprecipitation method, different metal ions are dissolved in water. Then, the metal ion solution is added to 2 M NaOH (pH 14) solution and stirred for 30 min at 100 °C. Finally, deionized water is used to wash the product and decrease the pH to near 7 [152]. Additionally, in the micro emulsion method, two organic and inorganic phases consisting of one or more cationic or anionic species are added together. The solution is stirred until the organic solvent has fully been removed. The product is washed with water and dried in vacuum. Finally, calcination is used to obtain the desired nano-platform product.

##### Application of Transition Metals in PDT

**Figure 5:**
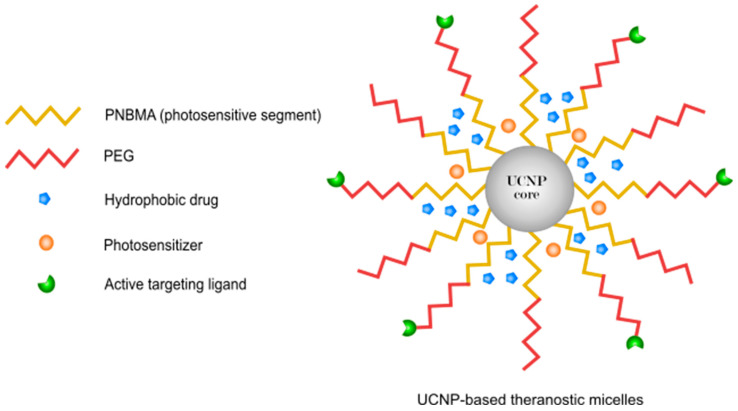
Targeted up-conversion nanoparticle-based micelles for simultaneous near infrared-controlled combination chemotherapy and PDT. Polyethylene glycol: PEG, and poly(4,5-dimethoxy-2-nitrobenzyl methacrylate): PNBMA (figure created with Inkscape).

Different nano-platforms such as NaYF_4_:Yb/Tm/Er [81] and NaYF_4_:Yb/Er [82] are reported to have up-conversion ability (Figure 5). For example, in 2016, Fujin Ai and co-workers [80] assembled the core–shell–shell biocompatible nano-platform NaGdF_4_:Yb/Nd@NaGdF_4_:Yb/Er@NaGdF_4_, which was loaded by platinum prodrugs [153]. This platform could be considered as up-conversion nanoparticles that are able to emit ultraviolet and visible light after near infrared irradiation [154,155]. This visible light can help the selected photosensitizer to generate singlet oxygen. In another project, Wang and co-workers [156] synthesized the magnetic nano-platform YbPO_4_:Er by using the solvothermal method. The nano-platform was able to convert near infrared light (980 nm) to visible light (450–570 nm). Doxorubicin was used as an anticancer drug to achieve synergistic effects from chemotherapy and PDT. This nano-platform successfully entered into human hepatocellular carcinoma cells and demonstrated low toxicity.

**Figure 6:**
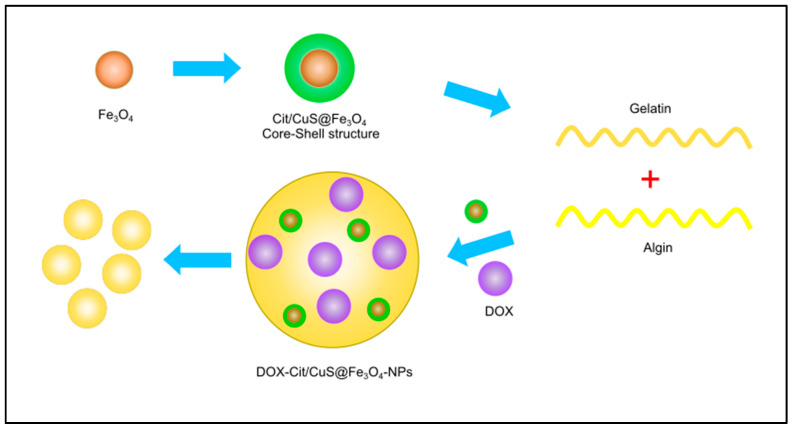
Schematic illustration of DOX-citric acid/CuS@Fe_3_O_4_. An example of transition metals utilized as nanocarriers. Doxorubicin: DOX (figure created with Inkscape).

Transition metals are considered promising nanocarriers to deliver therapeutic agents to cancer cells (Figure 6). In addition to their photosensitizing ability, they can also be used as nanocarriers, which is very interesting for these types of materials. For example, in 2011, Zhang and co-workers [35] prepared ZnO nano-rods containing daunorubicin as an anti-cancer drug during a one-step solid state reaction under ambient temperature. It was observed that the concentration of daunorubicin was significantly increased in human hepatocarcinoma cells (SMMC-7721cells), which demonstrates that ZnO nano-rods are not only good photosensitizers, but can also be considered as promising drug carriers for daunorubicin [157]. In other projects, Zhang and co-workers [32] developed a Cu_2−x_Se nano-platform for the treatment of malignant glioblastoma with near infrared PDT and chemotherapy by using doxorubicin as an anticancer drug. Infrared absorption of Cu_2−x_Se was around 1064 nm, and it was strong enough to penetrate deeply into the desired tissue. It was also able to efficiently degrade H_2_O_2_ and oxygen within the tumor to produce vast amounts of reactive oxygen species [158,159]. Other research groups have also reported nanoparticles that can be applied as both nanocarriers and photosensitizers such as citric acid/CuS@Fe_3_O_4_ [27], zinc(II) phthalocyanine [160], cyclometallated iridium (III) [31], silver nanoparticles [34], and NaYF4:Yb/Tm [38] All nano-platforms demonstrated good in vitro and in vivo therapeutic efficacy.

In some cases, nano-platforms are primarily used as nanocarriers. For instance, Imanparast and co-workers [77] prepared PEGylated hollow gold nano-platform as a carrier for the therapeutic drug mitoxantrone, which is both a photosensitizer and chemotherapy agent. Using the hollow gold nanoparticles had advantages such as biocompatibility and high stability [161]. Wang and co-workers [85] developed a layered double hydroxide [Mg_(1−x)_Al_x_(OH)_2_][A^n−^_x/n_]·zH_2_O as a cationic nanocarrier to deliver the anti-cancer prodrug *c*,*c*,*t*-[diamine-dichlorodisuccinato-platinum(IV)] and photosensitizer chlorin-e6 to improve the activity of cisplatin in cisplatin-resistant human cancer cells [162]. The release mechanisms used visible light irradiation and oxidation/reduction by which Ce and cisplatin were released from the layered double hydroxide.

#### 2.2.2. Silica

Different types of silica nanoparticles have been extremely widely used in drug delivery systems [163,164] due to their biocompatibility, high surface area, high stability, capability of surface modification, and controllable size. These abilities make mesoporous silica a perfect nano-platform for a variety of therapies such as a combination of chemotherapy and PDT [165].

##### Synthesis Routes of Silica

In general, the following three methods are employed for the synthesis of solid silica nanoparticles. Stöber’s method was discovered in 1968 and is among the most significantly used methods for the preparation of silica nanoparticles. In this method, different types of silicates such as tetraethoxysilane are mixed with ammonia, water, and ethanol to synthesize the requested silica nanoparticles. The concentration of solvents and silica additives can determine the size of the nanoparticles [166]. In the reverse micro-emulsion method, the spherical micelles are formed by adding a surfactant to an organic solvent that is transparent and thermodynamically stable. The preparation of silica nanoparticles occurs in the interface of the micelles [167]. In the chemical vapor deposition method, which is also called the high temperature flame decomposition method, precursors such as silicon tetrachloride are brought into the vapor phase to be prepared for nucleation [168,169].

##### Application of Silica in PDT

For example, in 2014, Fan and co-workers [78] successfully prepared a gadolinium/mesoporous silica core/shell nano structure to co-deliver hematoporphyrin and docetaxel as the photosensitizer and chemotherapeutic agents. The nano-platform was irradiated by near infrared and X-ray, which led to the complete elimination of the tumor by the synergistic effect of chemo, radio, and photodynamic therapies [170]. Yang and co-workers [88] prepared mesoporous silica nanoparticles that were doped by chlorin-e6. The structure of the silica matrix was changed from sphere to rod-like shapes due to the incorporation of chlorin-e6 into the matrix. This change was interesting because rod-like mesoporous silica was more efficiently taken up by cells. In this study, doxorubicin was utilized as the anti-cancer drug. In 2015, Yao and co-workers [91] synthesized mesoporous silica nanoparticles that were modified by PEGylated tetraphenylporphyrin zinc using the acid sensitive *cis*-aconitic anhydride bond. Silica pores can also be synthesized to have a positive charge if the pH is around 6.8. Having a positive charge increases cellular internalization, enhancing the efficacy of this nano-platform [171].

**Figure 7:**
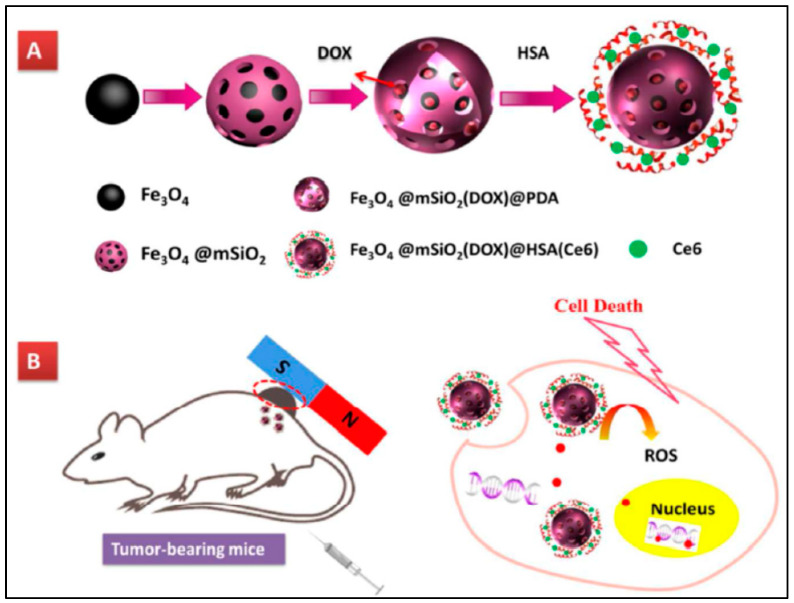
(**A**) A schematic illustration of the Fe_3_O_4_@mSiO_2_(DOX)@HSA(Ce6) synthesis process. (**B**) The schematic illustration of the application of Fe_3_O_4_@mSiO_2_(DOX)@HSA(Ce6) nano-platform in cancer therapy. Human serum albumin: HAS. Reproduced with permission from [92], ACS, 2018.

In 2018, Tang and co-workers [92] (Figure 7) successfully synthesized Fe_3_O_4_@mSiO@human serum albumin to act as a carrier for doxorubicin. They also used chlorin-e6 as the photosensitizer during this study. The nano-platform was irradiated by red light and utilized for the treatment of glioma cells. In 2015, Yang and coworkers [93] prepared mesoporous hollow silica-fullerene nanoparticles by the reverse micro-emulsion method. Doxorubicin was encapsulated into the inner cavity, and fullerene was incorporated in the shell to act as the photosensitizer. The ability of fullerene as a photosensitizer was increased in mesoporous hollow silica because the pores that formed silica shells can increase the interaction between oxygen and fullerene to generate singlet oxygen more efficiently. In 2016, Zhang and co-workers [94] designed mesoporous silica nanoparticles to deliver the chlorin-e6 photosensitizer and cisplatin prodrug to be used as a nano-platform for the treatment of A549R lung cancer cells. Cisplatin prodrug was conjugated to silica by the β-cyclodextrin-grafted polyethylenimine linker. The nano-platform was irradiated by red light (660 nm), and it was observed to give a half-maximal inhibitory concentration (IC_50_) value was around 0.53 μM, which was much lower than that of cisplatin.

#### 2.2.3. Graphene

Graphene is a 2D material that is an allotrope of carbon consisting of a single layer of atoms arranged in a two-dimensional honeycomb lattice. Graphene has attracted tremendous research interest in recent years due to its exceptional properties. The scaled-up and reliable production of graphene derivatives such as graphene oxide (GO) and reduced graphene oxide (rGO) offer a wide range of possibilities to synthesize graphene-based functional materials for various applications [172].

Tremendous efforts have been made to develop synthetic methods for graphene to achieve high yields of production. Methods to make graphene can be generally classified as bottom–up and top–down approaches. The bottom–up approach involves the direct synthesis of graphene materials from carbon sources such as the chemical vapor deposition (CVD) [173] or plasma enhanced CVD (PECVD) [174]. In comparison with the bottom–up approaches, the top–down approaches are advantageous in terms of high yields, solution-based process ability, and ease of implementation, which have been demonstrated by means of intercalation, chemical functionalization, and/or sonication of bulk graphite. The first observation of exfoliated graphite dates back to 1840 by Schafhaeutl, when H_2_SO_4_ was used for the intercalation [172]. Since then, a number of chemical species have been found to form intercalated compounds with graphite [175,176]. Further attempts by combining the intercalation and sonication have realized the isolation and dispersion of graphene sheets by using intercalates such as *N*-methyl-pyrrolidone (NMP) [177] and sodium dodecylbenzene sulfonate (SDBS) [178] in non-aqueous and aqueous solutions, respectively.

##### Application of Graphene in PDT

**Figure 8:**
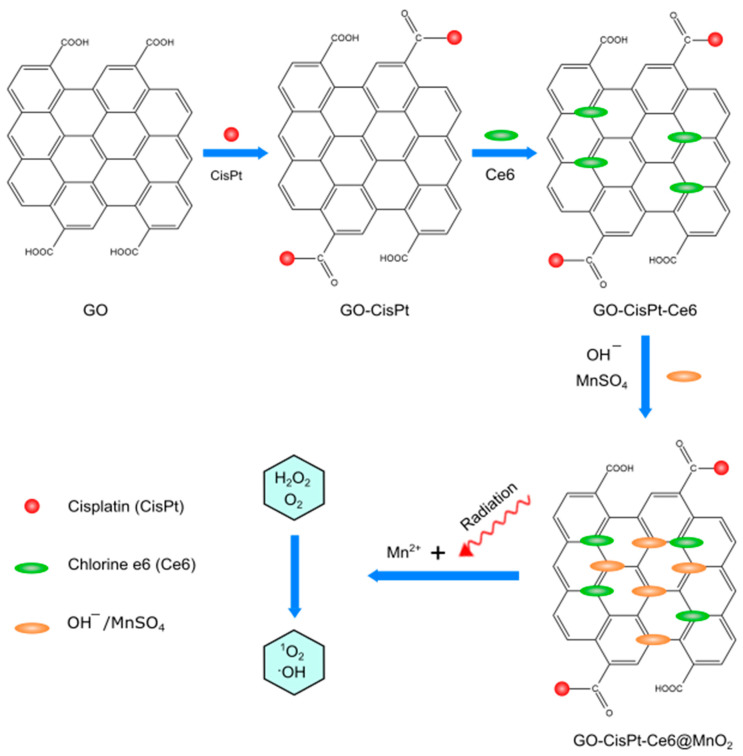
Preparation routes of Go-CisPt-Ce6@MnO_2_. Graphene oxide: GO (figure created with Inkscape).

In 2014, Jiang co-workers [104] used graphene oxide to deliver Hypocrellin A (photosensitizer). They observed that after loading of Hypocrellin A on graphene oxide, the anticancer activity of Hypocrellin A was decreased. Therefore, they utilized 7-ethyl-10-hydroxycamptothecin (SN-38) as the second chemotherapeutic agent to solve the problem. SN-38 was co-loaded on GO (Hypocrellin A/SN-38/GO) by hydrogen bond and π–π stacking interaction to combine PDT and chemotherapy synergistically for an antiproliferative effect. In 2020, Zhou and co-workers [105] found a way to reduce tumor hypoxia by the self-production of O_2_ and decrease intracellular GSH amounts to improve PDT and chemotherapy. They designed a nanosheet based on MnO_2_-doped GO to load CisPt and chlorin-e6 simultaneously. They found that, in addition to MnO_2_ ability to decompose H_2_O_2_, it also decreases GSH levels in cancer cells (Figure 8).

**Figure 9:**
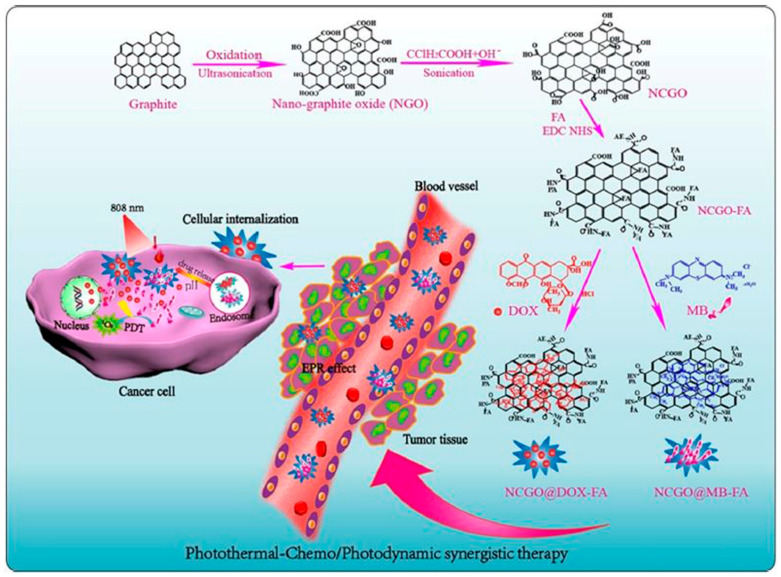
Performance of the versatile nano-platforms NCGO@DOX-FA and NCGO@MBFA targeted drug delivery systems for photothermal-chemo/photodynamic synergetic therapies. Folic acid: FA, and Methylene blue: MB. Reproduced with permission from [107], ACS, 2019.

In 2020, Vinothini and co-workers [106] decorated a reduced GO surface with magnetic nanoparticles as a new nano-platform that loaded with camptothecin (CPT) chemodrug and 4-hydroxy coumarin (4-HC) photosensitizer (365 nm laser irradiation of 20 mW/cm^2^). The combined treatment indicated exceptional cell apoptosis and antitumor activity. In 2019, Liang and co-workers [107] (Figure 9) fabricated a targeted nano system (GO-Folate) with an ultrahigh surface area by Hummers’ method, which is loaded by DOX and methylene blue (MB) via π−π stacking and hydrophobic or electrostatic interactions with high-load content. This nano-platform triggered DOX and MB release by heat and an acidic pH in tumor environments. In 2017, Zhao and co-workers [179] used a macrophage transferring system (TAM), which effectively enhances the effect of cyclophosphamide (CTX)-loaded 2-(1-hexyloxyethyl)-2-devinyl pyropheophor-bidealpha (HPPH)-coated PEG nano-graphene oxide [GO(HPPH)-PEG] by increasing its infiltration into tumors (670 nm, 70 J/cm^2^).

#### 2.2.4. Liposomes

Liposomes have an aqueous solution core surrounded by a hydrophobic membrane in the form of a lipid bilayer; hydrophilic solutes dissolved in the core cannot readily pass through the bilayer. Hydrophobic chemicals associate with the bilayer. A liposome can hence be loaded with hydrophobic and/or hydrophilic molecules. To deliver the molecules to a site of action, the lipid bilayer can fuse with other bilayers such as the cell membrane, thus delivering the liposome contents. Because of their structure as well as their high loading capacity and ability to be modified, liposomes have been significantly used as nanocarriers for different types of drugs. Their unique structure allows liposomes to be accumulated in the tumor site efficiently and after modification, they can exhibit a long time plasma half-life, which is important for tumor uptake. Hence, different types of photosensitizers and anti-cancer agents can be loaded into modified liposomes simultaneously for use in both PDT and chemotherapy [180].

##### Synthesis Routes of Liposomes

Although different types of methods have been reported for the preparation of liposomes, all of them commonly consist of the following steps: (a) drying down lipids from organic solvents; (b) dispersing the obtained lipid in aqueous media; and (c) purifying the resultant liposome [181]. The sonication method is the most significantly used method for multilamellar vesicles. In this method, a probe or bath sonication is used to prepare liposomes under a passive atmosphere [182]. Additionally, in the solvent dispersion method, lipids are dissolved into an organic solvent to prepare an organic phase. Then, the organic phase is added gradually to an aqueous solution of the materials that are going to be encapsulated, at more than 50 °C. Finally, liposomes can be created by complete evaporation of the organic phase [183]. The freeze-thawed method uses multiple cycles of a rapidly frozen and slowly thawed solution of liposomes, as the name implies. First, materials that are used to prepare liposomes are separated by sonication for a short time. Then, the system will be rapidly frozen and slowly thawed to allow for unilamellar vesicles to be fused and created [184]. The extrusion method is another technique where the liposome suspension is passed through a membrane filter of a defined pore size. An extruder, a machine equipped with a pump that pushes fluids through the membranes, can be employed to accomplish the extrusion process. Various parameters of the extrusion procedure such as applied pressure, number of cycles, and pore size have been found to influence the mean diameter and size distribution (polydispersity) of the liposomes produced [185].

##### Application of Liposome in PDT

In 2003, Snyder and co-workers [95] used 2-[1-hexyloxyethyl]-2-vinyl pyropheophorbide as the photosensitizer and liposomally encapsulated doxorubicin as an anti-cancer drug. The liposome was used for the treatment of murine colon 26 tumors, which showed improvement in accumulation and selectivity due to enhanced vascular permeability by liposome. In 2018, Lee and co-workers [96] encapsulated doxorubicin into human serum albumin/chlorin-e6 as a photosensitizer into ultrasound-triggered microbubbles that were prepared by different mixtures of lipids (DSPC: DSPE-PEG2k-NHS). The researchers used sonoporation, which is the use of sound to modify the permeability of the cell membrane during the treatment, to convert microbubbles to liposomes and enhance the efficiency of PDT. This result revealed that doxorubicin and chlorin-e6 were delivered into the cells and penetrated the tumor tissues with the aid of local ultrasound irradiation. Moreover, both drugs can be delivered by sonoporation, and the mechanical effects of ultrasound irradiation into deep tumor sites where the drug has difficulty reaching from the bloodstream. In 2018, Li and co-workers [97] prepared a light sensitive liposome through the combination of indocyanine green-octadecylamine and doxorubicin as the photosensitizer and chemotherapeutic agent. The surface of the liposome was functionalized by epidermal growth factor receptor-2 (Her2) antibodies, and it was irradiated by near infrared light (808 nm) for the treatment of MCF-7 breast cancer cell lines. In 2019, Yang and co-workers [98] encapsulated lipophilic IR780 (photosensitizer) and hydrophilic tirapazamine (anti-cancer agent) into a liposome for the treatment of hypoxic malignant tumor cells. The system was irradiated by near infrared (808 nm) light. IR780 could generate a hypoxic microenvironment, which is very suitable for tirapazamine to perform well and cause DNA double-strand breaks and chromosome aberrations.

#### 2.2.5. Dendrimers

Dendrimers consist of highly branched molecules that are designed three-dimensionally. Examples include poly(propylene imine), polyesters, peptide dendrimers, triazine dendrimers, and polyamidoamine (PAMAM), which have great potential in biomedical applications due to their high loading efficiency and low toxicity [186,187,188,189,190]. Anti-cancer drugs and photosensitizers can be encapsulated within the dendrimer or conjugated to surface molecules such as acyl hydrazone or ester groups [191,192,193].

#### 2.2.6. Preparation Methods of Dendrimers

There are two methods that are mainly used for the preparation of dendrimers termed as the divergent and convergent methods. In the divergent method, the synthesis starts with the core of the dendrimer and arms are added gradually to prepare the final desired 3-dimensional form of the desired dendrimer, and in the convergent method, the arms are initially prepared and then subsequently attached to the core to create the desired final form [194,195].

##### Application of Dendrimers in PDT

In 2016, Liu and co-workers [99] developed a dendritic poly(ethylene glycol) copolymer that was conjugated to porphyrin (photosensitizer) by a disulfide linker. Doxorubicin was conjugated to the dendrimer utilizing the same disulfide linker to be glutathione responsive due to glutathione’s ability to reduce the disulfide bond. The nanocarriers showed higher loading efficiency and cellular uptake than the linear co-polymer. The dendrimer was irradiated by a visible light emitting diode (LED) to exhibit a great potential for PDT and chemotherapy [179].

#### 2.2.7. Polymers

The ability of polymer micelles to self-assemble due to their amphiphilic nature have gained much interest in drug delivery [196,197,198,199,200,201]. Self-assembled polymeric micelles exhibit nano sized spherical structures, high thermodynamic stability, and biocompatibility.

As a new category of organic theranostic agents, semiconducting polymer nanoparticles (SPNs) have gained growing attention due to their diversified optical properties [202]. Moreover, structural modification of precursor polymers has led to SPN-based phototherapeutics. These agents are able to convert photo-energy to heat or reactive oxygen species for PDT or photo thermal therapy [203,204].

##### Main Synthesizing Methods

Synthetic methods are generally divided into two categories: step-growth polymerization [205] and chain polymerization [206]. The essential difference between these two processes is that in chain polymerization, monomers are added to the chain one at a time only, whereas in step-growth polymerization, chains of monomers are combined with one another directly. Step-growth polymerization can be further divided into polycondensation and polyaddition [207,208].

##### Application of Polymers in PDT

In 2018, Gao and co-workers [108] developed a polymeric micelles (methoxypolyethylene glycol (mPEG) and poly(β-benzyl-l-aspartate) (PBLA)) encapsulating DOX (chemotherapeutic) and zinc(II) phthalocyanine (ZnPc) as the photosensitizers for dual therapy. Doxorubicin and ZnPc were conjugated to the polymer by an acid-labile hydrazone (pH sensitive) linker and a redox-responsive disulfide linker. Tests revealed that with increasing glutathione (GSH) levels, the disulfide linkers were cleaved and ZnPc moieties were released, which diffused out from the dialysis membrane. In 2018, Li and co-workers [209] designed stimuli-responsive nanoparticles based on an amphiphilic co-polymer containing arylboronic ester (BE)-modified with an amphiphilic co-polymer (mPEG-PBAM). The prepared polymers formed micelles and were loaded with DOX and hematoporphyrin (Hp) [109] as a PS (light irradiation: 635 nm, 5 mW/cm^2^). After irradiation, the BE part of the polymer was cleaved due to ROS generation. ROS oxidizes the hydrophobic segment, making it hydrophilic and destabilizing the structure. In 2016, Li and co-workers [110] investigated micellar nanoparticles based on methoxy-poly ethylene glycol-poly caprolactone (mPEG-PCL), which encapsulated docetaxel (DTX) [210] and NIR dye-IR820 (indocyanine green derivative; irradiation: 808 nm, 2.5 W/cm^2^) for the synergistic therapy of breast cancer. In 2015, He and co-workers [211] reported a nano-micellar carrier based on a coordination polymer (NCP) loading a high amount of cisplatin (25%), and phospholipid-porphyrin (pyrolipid) was also used as a photosensitizer for combined therapy (irradiation at 670 nm LED, 100 mW/cm^−2^). At sufficiently high pyrolipid loadings (when its lipid layers were intact), the fluorescence of pyrolipid molecules will self-quench due to their proximity to each other. Therefore, Triton X-100 was added to the nano-platform to disrupt the lipid layer, and NCP@pyrolipid could efficiently generate ^1^O_2_, which was confirmed by fluorescence intensity (singlet oxygen sensor green). In 2016, Chunbai He and coworkers [111] evaluated immunogenic nanoparticles to enhance the antitumor efficacy using a checkpoint inhibitor such as antibodies to inhibit the PD-1/PD-L1 axis for colon cancer immunotherapy. This nanoparticle is based on a NCP (1,2-distearoyl-sn-glycero-3-phosphocholine, cholesterol, 1,2-distearoyl-sn-glycero-3-phosphoethanolamine polyethylene glycol 2000) carrying oxaliplatin as a chemo-drug and the photosensitizer pyrolipid (irradiation at 670 nm LED, light dose of 180 J·cm^−2^ given with 100 mWcm^−2^) for effective co-therapy that stimulated an immune response.

In 2017, Zhu and co-workers [212] prepared an amphiphilic polyprodrug of poly(*N*,*N*-dimethylacrylamide-*co*-eosin)-*b*-poly camptothecin, which were assembled into hybrid nanoparticles by oleic acid-stabilized NaYF4:Yb/Er to activate the eosin under a NIR laser irradiation (980 nm laser, 1.5 W/cm^2^). In 2016, Ruan and co-workers [112] synthesized a pH-responsive polymeric micelle based on mPEG-PASP-benzaldehyde (PASP: polyaspartic acid) conjugated with DOX and encapsulated with NIR photosensitizer 4,4-difluoro-4-bora-3a,4a-diaza-sindacene (BODIPY) for both bioimaging and PDT (635 nm, 20 mW cm^−2^) [213]. BODIPY has many attractive properties such as high ratios of light–dark toxicity and resistance to photobleaching. In 2018, Yi and co-workers [113] designed a dual-delivery micelle based on amphiphilic polymeric prodrug poly(ethylene glycol)-b-poly(5-mthyl-5-propargyl-1,3-dioxan-2-one)-g-paclitaxel (PMP) to load a red induced emission fluorogen photosensitizer, TB (white light, 100 mW·cm^−2^), and a chemodrug, PTX for synergistic PDT and chemotherapy. In 2018, Shi and co-workers [114] developed a PEGylated prodrug of DOX using thioketal linkage and cRGD (cyclo-arginine-glycine-aspartic acid-d-phenylalanine-cysteine) peptide (RPTD) as a ROS-sensitive nanoparticle that was encapsulated by the photosensitizer hematoporphyrin (HP) (633 nm at 100 mW/cm^2^) via π–π stacking interactions. The release of doxorubicin was ROS-responsive from the prepared nanoparticles because of the break of the thioketal linker. In 2009, Peng and co-workers [79] designed functionalized micelles based on a chlorin-core star-shaped block co-polymer by a lyophilization–hydration method. This chlorin-core star-shaped block co-polymer acts as a nano-photosensitizing agent (7 J/cm^2^ irradiation) by encapsulating a promising antitumor drug 7-ethyl-10-hydroxy-CPT (SN-38). In 2019, Zhen and co-workers [115] reported a novel micelle based on the polymeric prodrug poly(ethylene glycol)-b-poly(5-mthyl-5-propargyl-1,3-dioxan-2-one)-g-paclitaxel, which was loaded with a NIR fluorophore as a photosensitizer that demonstrates a strong NIR emission for imaging applications and charge transfer properties for multidrug resistance tumor. In 2019, Zhu and co-workers [116] prepared a PDT-induced hypoxia-responsive drug delivery system by self-assembling amphiphilic polyethylenimine-alkyl nitroimidazole (PA) and hyaluronic acid-chlorin-e6 (660 nm, 10 mW/cm^2^) to load tirapazamine (TPZ) as a bioreductive chemodrug [214]. TPZ can be changed to a toxic chemodrug via single-electron reduction in hypoxic environments [215]. In 2013, Conte and co-workers [117] investigated a unique core-shell carrier with diblock (AB) and triblock (ABA) structures based on amphiphilic block co-polymers poly(ε-caprolactone) (PCL = B) and poly(ethylene oxide) (PEO = A) for co-delivery of the lipophilic chemodrug docetaxel (DTX), and the second generation photosensitizer ZnPc (610 nm) by the melting/sonication method to treat an animal model of orthotopic amelanotic melanoma.

In 2019, Cui and co-workers [49] designed semiconducting polymer nanoparticles (SPNs) based on a light-responsive photodynamic backbone. The SPNs were grafted with poly (ethylene glycol) (PEG) and conjugated with the chemodrug molecules via hypoxia-cleavable linkers [216]. These SPNs efficiently produced ^1^O_2_ under NIR photo-irradiation and activated its chemotherapeutic action in a hypoxic tumor environment, leading to cell death.

The layer-by-layer (LbL) assembly technique [217] is an effective way to produce thin-film materials, which can control the configuration and specific functions of materials such as polymers using external stimuli. These kinds of blocks can be designed into multilayer thin films by direct alternating deposition, or by employing the preassembly of building blocks. In particular, LbL films provide a useful platform for combining chemotherapy and PDT. For example, in 2016, Fan and co-workers [218] prepared tellurium-containing photoresponsive polyelectrolyte multilayer films by LBL assembly of a tellurium-containing two polymer. The polymers were (piperazine and PEG). They also used indocyanine green (ICG) and porphyrin as photosensitizers and poly(styrenesulfonate) as an anionic building block to make the film stronger and stable. The production of singlet oxygen oxidizes tellurium to a high valence state (Te = O) on the polymer backbone, which makes the micelles more hydrophilic, and facilitates the release of the loaded cargo from the micelles. In 2018, Wang and co-workers [118] investigated unique multifunctional polysaccharide-based nanoparticles by LbL self-assembly using hydroxyethyl chitosan (HECS) and aldehyde-functionalized hyaluronic acid (AHA), which were stabilized through Schiff’s base bond and electrostatic interactions. These particles were loaded with DOX and pro-photosensitizer 5-aminolevulinic acid (635 nm light irradiation, 0.2 W).

In the last decade, utilization of covalent-organic polymers (COPs) as therapeutic agents [219] has received substantial attention in clinical fields. COPs can covalently cross-link different types of organic molecules to form organic network configurations. For example, in 2018, Wang and co-workers [119] evaluated a new class of COPs using cross-linking of mesotetra(p-hydroxyphenyl) porphine (THPP) as a photosensitizer (using a 660-nm LED light at a power density of 5 mW·cm^−2^) to a chemo pro-drug, cis-platinum (Pt). Polyethylene glycol was conjugated to this pro-drug (THPP-Pt-PEG COPs) by the one-pot reaction. THPP-Pt-PEG COPs could be stored in a lyophilized form and occur as stable nanoparticles in aqueous solution. Upon intravenous injection, the COPs demonstrated long blood circulation time, tumor accumulation, and after injection of COPs into mice, vascular perfusion and largely relieved tumor hypoxia, which are all favorable for photodynamic treatment. In 2018, Wang and co-workers [220] presented a new type of pH-responsive COPs by using acryloyl meso-tetra(*p*-hydroxyphenyl) porphine (acryloyl-THPP) as a photosensitizer (660 nm, 5 mW·cm^−2^) and the pH-responsive crosslinked biodegradable β-amino esters (BAEs), which are terminated by PEG shell (THPP-BAE-PEG COPs). These COPs encapsulated DOX into their porous structure.

There are other nano-platforms that utilize polymers to make specific components or encapsulate them in a proper shell. For example, in 2017, Wang and co-workers [221] demonstrated an effective nanocarrier based on phospholipid/pluronic F68 complex nanocores and pullulan (polysaccharide) shells to carry IR780 (a near-infrared dye) [222,223] and paclitaxel (PTX) [224]. Additionally, pullulan acts as a natural ligand for the asialoglyco protein receptor (ASGPR) [224], which is often overexpressed by HCC cells. In 2018, Liu and co-workers [225] reported a light-responsive porphyrin-dextran-based polymeric DOX conjugate to control DOX release through ROS-cleavable linker combined with PDT. In 2009, Khdair and co-workers [226] constructed aerosol OT (AOT)-alginate nanoparticles for the co-delivery of DOX and methylene blue (a photo activated dye by 665 nm wavelength) in drug-resistant NCI/ADR-RES cells (a multidrug-resistant cell line in ovarian cancer). In 2008, Hongrapipat and co-workers [227] evaluated the biological activities of the anticancer drug SOS thiophene (SOS) and M_chlorin-e6_ (650 nm at 3.0 mW/cm^2^) in the form of Fab′-targeted HPMA co-polymer-drug conjugates (Fab′ from OV-TL16 antibodies matching to CD47) against OVCAR-3 cells, which indicated a very strong synergism. In 2016, Dong and co-workers [228] fabricated a DOX-loaded protein/polymer coated-up conversion nanosystem including a UCN core (NaYF4:Yb/Er), folic acid-bovine serum albumin−poly(ε-caprolactone) (FABSA-PCL) as an amphiphilic bioconjugate shell, and ZnPc as a photosensitizer (980 nm laser at a power density of 1.0 W cm^−2^). In 2010, Khdair and co-workers [226] improved the anticancer efficiency of DOX in combination with the PS methylene blue (50 J/cm^2^ dose of non-coherent light at 665 nm) [229] in a tumor model. These two drugs were encapsulated in surfactant-polymer hybrid nanoparticles, which were synthesized by an anionic surfactant, aerosol-OT™ (AOT), and a polysaccharide polymer, sodium alginate. In 2019, Ren and co-workers [120] fabricated hyaluronic acid-chlorin-e6 (DOX) as an enzyme/pH responsive nanoparticle. In this nanoparticle, HA is combined with a highly effective photosensitizer (chlorin-e6) by adipicdihydrazide (ADH) as a linker. Chlorin-e6 is a second generation photosensitizer that is able to be activated by NIR light and is used for PDT [230]. In 2014, Shi and co-workers [121] synthesized a DOX-conjugated onto poly(ethyleneimine) (PEI)-fullerene (C60–PEI–DOX) to facilitate photosynamic therapy and chemotherapy in one system as well as evaluate its synergistic effect on cancer cells. C60 has been introduced as a nanocarbon material with exceptional photochemical (532 nm laser, 100 mW·cm^−2^) and physical properties. They used a hydrazone linker to make doxorubicin’s release pH sensitive. Compared with free DOX in an in vivo murine tumor model, C60–PEI–DOX afforded higher antitumor efficacy without obvious toxic effects to normal organs due to its good tumor targeting efficacy and the 2.4-fold greater amount of DOX released in the tumor than in the normal tissues. In 2018, Hu and co-workers [122] prepared oxygen-generating (CDM) nanoparticles by assembling chlorin-e6 (660 nm, 100 mW/cm^2^), DOX, and manganese dioxide (MnO_2_) with poly (ε-caprolactone-co-lactide)-b-poly (ethylene glycol)-b-poly (ε-caprolactone-colactide) for breast cancer therapy. MnO_2_ caused the breakdown of excessive endogenous H_2_O_2_ to produce O_2_ inside the tumors to relieve tumor hypoxia. With enhanced oxygen generation, the PDT effect was significantly improved under laser-irradiation. More importantly, this effect, together with that of DOX, was able to dramatically promote the combined chemotherapy-PDT efficacy of CDM NPs in an MCF-7 tumor-bearing mouse model.

In 2015, Wang and co-workers [123] prepared a new smart nanoparticle (pH-sensitive and NIR light triggered) based on UCNP-loaded (NaYF_4_:Yb, Er) folate-conjugated polymeric (dextran) lipid vesicles (UFPLVs) that carried DOX and merocyanine 540 (MC540) as a photosensitizer (980 nm, 2.5 W cm^−2^). In 2017, Yu and co-workers [124] used human serum albumin (HSA) as an effective nanodrug carrier for the delivery of gemcitabine (Gem) and pyropheophorbide-a (670 nm light, 10 mW/cm^2^) for pancreatic cancer.

Albumin is a versatile protein with a unique structure that can be conjugated to hydrophobic and hydrophilic components [231,232]. In 2017, Zhang and co-workers [233] prepared a DOX-loaded magnetofluorescent carbon quantum dots (FeN@CQDs) into polymer nanospheres (PEG) with magnetic and photoluminescent features using a low-cost and environmentally friendly one-pot hydrothermal method using iron crosslinked chitosan components (Ch-Fe-CL) [234]. Riboflavin (Rf) was grafted onto the surface of magnetic CQDs to be useful in triggering PDT under NIR light, which significantly improved tissue penetration. In 2017, Zhang and co-workers [125] fabricated a zinc phthalocyanine (8.12 mW/cm^2^) and DOX-loaded pH-sensitive four-armed star co-polymer nanocarrier, [methoxy-poly(ethylene glycol)-poly(2-(*N*,*N*-diethylamino)ethyl methacrylate)-poly(ε-caprolactone)]4-zinc β-tetra-(4-carboxyl benzyloxyl)phthalocyanine (PDCZP) that showed better in vitro and in vivo anticancer effects under lighting on MCF-7, SW480, and HepG2 cells and the murine hepatocellular carcinoma H22 mode.

#### 2.2.8. Metal–Organic Frameworks

Nanoscale metal organic frameworks are different types of hybrid porous nanomaterials that can be prepared by the coordinated interaction of metal ions and bridging ligands. Metal–organic frameworks have great potential in drug delivery systems due to their large surface area, high porosity, and modifiable surface chemistry [235]. Metal–organic frameworks have also been used in PDT to provide a synergistic effect for cancer therapy. For example, Zr_6_ clusters coordinated with terephthalic acid to form UiO-66, which has microporous cages and excellent stability, so this UiO-66 can be considered as a suitable candidate for drug loading [236,237].

##### Preparation Method of Metal–Organic Frameworks

In the solvo-thermal method, metal and organic precursors are added to an organic solvent and are stirred at room temperature until a clear solution is formed. Then, the homogenous mixture is transferred to a Teflon-lined autoclave and heated for 12 or 24 h. Finally, the desired product is separated via centrifugation or filtration. These MOFs can be modified by different organic ligands in the next levels [238,239].

##### Application of Metal–Organic Frameworks in PDT

**Figure 10:**
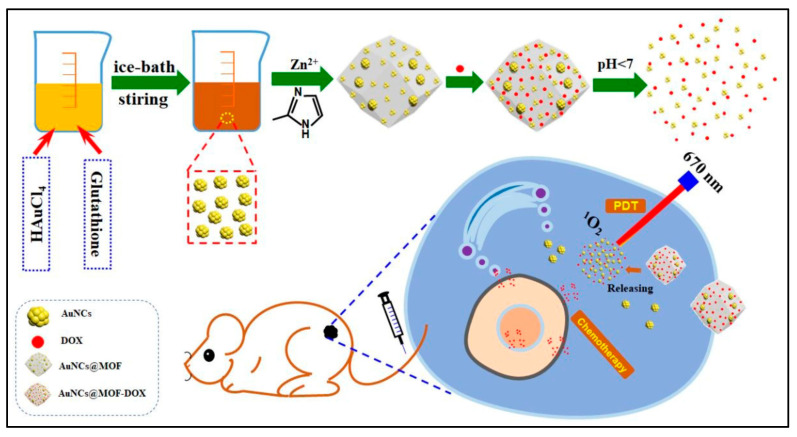
The synthesis process of AuNCs@MOF-DOX nanoprobes and their application in pH-responsive PDT and the chemotherapy of breast cancer. Doxorubicin: DOX. Reproduced with permission from [128], The Royal Society of Chemistry, 2020.

In 2019, He and co-workers [75] designed UiO-66 metal–organic frameworks (UiO-66-H/N_3_ NMOFs), and bioreductive banoxantrone (AQ4N), which was anchored to the nanocarriers by a phosphate ion-sensitive bond. Photosensitizers such as photochlor (HPPH) and azide were anchored to UiO-66 by the solvo-thermal method [240]. Moreover, PEGylating was utilized to improve the stability of nanocarriers. The porosity of the NPs is well-suited for the encapsulation of AQ4N to protect the bio-reductive prodrug from degradation during circulation. In this system, AQ4N release is demonstrated to be phosphate ion-sensitive. Both in vitro and in vivo studies revealed that the O_2_-depleting (consuming) PDT process does indeed aggravate intracellular/tumor hypoxia that activates the cytotoxicity of AQ4N through a cascade process, consequently achieving PDT-induced and hypoxia-activated synergistic therapy. Benefiting from the localized therapeutic effect of PDT and hypoxia-activated cytotoxicity of AQ4N, this hybrid nanomedicine exhibits enhanced therapeutic efficacy with negligible systemic toxicity, making it a promising candidate for cancer therapy. In 2020, Zhang and coworkers [128] (Figure 10) used ZIF-8 as metal–organic framework as a carrier to deliver Au and doxorubicin to achieve the synergistic effect of photodynamic therapy and chemotherapy. Under irradiation with a 670 nm laser, a large amount of singlet oxygen was generated, and the release rate of DOX increased to 77.1% at a pH value of 5.5. After using the combination therapy, all tumors were disappeared while single therapy could only inhibit tumors partially.

#### 2.2.9. Biological Nanocarriers

Among the different types of cell membranes, red blood cell membrane and its derivatives are the most affordable and biocompatible biological carriers that have been used to coat nanocarriers as biomimetic agents for various applications [241].

##### Preparation of Red Blood Cells Membranes-Derived Vesicles

Membrane-derived vesicles from red blood cells are used to prepare red blood cells for drug delivery systems. The first ones can be divided into two steps: hypotonic treatment and sequential extrusion. Briefly, fresh blood, which is obtained from an organism, should be centrifuged at 4000 rpm to collect red blood cells. Then, the collected red blood cells are mixed with phosphate buffer saline and remain to release the intracellular components of red blood cells following centrifugation to remove hemoglobin. The final step is utilizing an extruder to obtain the optimum size of the red blood cells [242,243].

##### Application of Biological Nanocarriers in PDT

In 2018, Pei and co-workers [100] developed red blood cell nanoparticles to deliver reactive oxygen species-responsive paclitaxel dimer and tetraphenylchlorin to cancer cells. It was observed that dimers can increase the loading of paclitaxel into red blood cells. The system was irradiated by visible light (638 nm) to generate reactive oxygen species, which caused paclitaxel to be released [244]. In 2018, Luo and co-workers [101] introduced hybrid protein oxygen carriers consisting of hemoglobin and albumin, which were attached together by disulfide reconfiguration. Doxorubicin and chlorin-e6 were loaded into the nano-hybrid. The ability of hemoglobin to carry oxygen provided a benefit in multiple ways. This feature led to the downregulation of the expressions of multidrug resistance 1 (MDR1), hypoxia-inducible factor-1α (HIF-1α), and P-glycoprotein (P-gp), which further breaks hypoxia-induced chemoresistance and interestingly helps chlorin-e6 to generate more ROS. In 2017, Wan and co-workers [126] fabricated nano-scaled red blood cells consisting of oxyhemoglobin and the gas-generating agent ammonium bicarbonate to deliver indocyanine green and doxorubicin as the photosensitizer and anti-cancer agent, respectively, for the treatment of breast cancer. After irradiation by 808 nm laser, oxyhemoglobin decomposed into CO_2_ and NH_3_, leading to the release of doxorubicin. It was observed that this nanocarrier could facilitate breast cancer treatment and suppress metastases by the combination of PDT and chemotherapy.

#### 2.2.10. Nano Emulsions

Nano emulsions are thermodynamically stable nanoparticles whose size (20–200 nm) and shape make them different from conventional emulsions. They consist of two immiscible liquids that are mixed by different types of surfactants.

##### Synthesis Routes of Nano Emulsions

There are several methods for the preparation of nano emulsions. However, all methods can be classified into two main methods: high-energy emulsification such as stirring, ultrasonic emulsification, high-pressure homogenization, micro-fluidization, and low-energy emulsification such as phase inversion temperature, emulsion inversion point, and spontaneous emulsification [245]. In the high-pressure homogenization method, a high pressure homogenizer/piston homogenizer is used to prepare a nano-emulsion with a particle size lower than one nanometer [246]. Additionally, in the micro-fluidization method, a micro-fluidizer, which uses high pressure to produce a very fine particle with a size range of 150–170 nm, is used. The process is a repeated procedure of forcing materials to pass through the interaction chamber to prepare a uniform nano emulsion [247]. The spontaneous emulsification method consists of three steps to prepare a nano emulsion. In the first step, an organic solution containing the oil and surfactants is prepared. In the next step, the organic phase is injected into the aqueous phase. Finally, the organic phase is removed by evaporation to prepare a nano emulsion [248].

##### Application of Nano Emulsion in PDT

In 2018, Maria Candido and co-workers [6] prepared nano emulsions to encapsulate hydrophobic chloroaluminum phthalocyanine as a photosensitizer and doxorubicin as an anti-cancer agent. The nano emulsion was able to increase the water solubility of chloroaluminum phthalocyanine, which was crucial to the efficiency of the drug delivery system. Visible laser light was used to irradiate the nano emulsion, which caused the photosensitizer to generate reactive oxygen species for the treatment of 4T1 breast cancer cells. It was observed that the cell viability was less than 10% when a combination of PDT and chemotherapy was applied. Therefore, this nano-platform can be considered as a promising method of treatment for breast cancer [249,250]. In 2021, Chang and coworkers [251] prepared porphyrin-lipid nano emulsions and loaded doxorubicin, PTX (3.1 wt%), and porphyrin (18.3 wt%) efficiently into PLNE-PTX, forming spherical core–shell nano emulsions. Combination therapy inhibited tumor growth (78%) in an additive manner compared with monotherapy PDT (44%) or chemotherapy (46%) 16 days post-treatment.

### 2.3. Targeting Strategy

**Figure 11:**
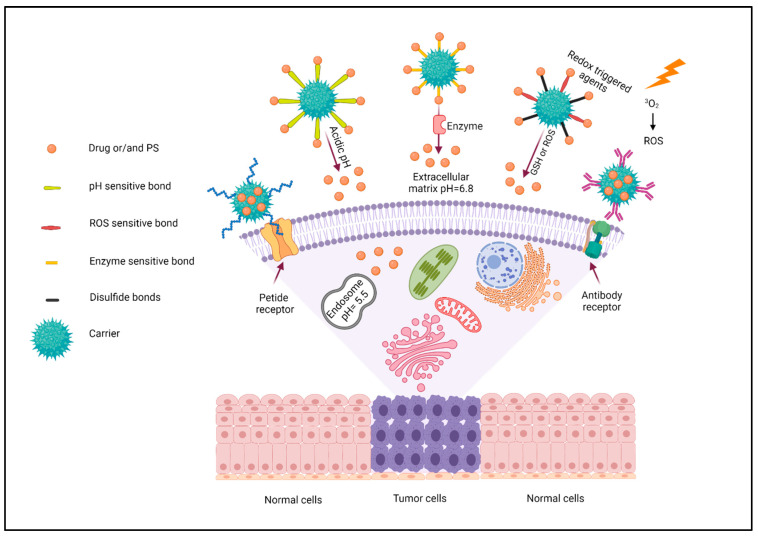
Schematic illustration of various targeting strategies in PDT (figure created with BioRender.com, access 17 January 2021).

Various strategies such as pH triggered, enzyme triggered, chemical targeting agents, biological targeting agents, and redox triggered agents have been used as targeting strategies to deliver drugs to a specific part of the body (Figure 11). Photosensitizers can be loaded on nanocarriers and targeted to a specific tissue using these various means. These targeting materials can be added to the nanocarriers to increase the accumulation and efficiency of drugs and decrease the side-effects and frequency of dosage taken by the patient [252,253,254,255,256].

#### 2.3.1. pH Triggered

The important point in this strategy is the physiological differences in pH. Internal cellular structures such as lysosomes have reduced pH (~pH = 4) and certain cancerous cells are more acidic than normal healthy cells. Materials can exploit this feature and are considered pH responsive and upon reaching a lower pH environment, this trigger should be able to collapse, swell, or change the nanocarrier according to the change in the pH of their environment [257,258,259].

For example, Gao and co-workers [108] introduced a co-polymer consisting of methoxypolyethylene glycol (mPEG) and poly(β-benzyl-l-aspartate) (PBLA) to encapsulate zinc(II) phthalocyanine and doxorubicin via an acid-labile hydrazone linker for HepG2 human hepatocellular carcinoma cells. In 2014, Shi and co-workers [121] prepared a nano-platform consisting of poly(ethyleneimine), which was conjugated to fullerene (C60). Doxorubicin was conjugated to the nano-platform via pH-sensitive hydrazine [260]. Then, the hydrazine bond was again used to conjugate doxorubicin to the surface of a lipid vesicle [123]. In all three studies, researchers concluded that this strategy induces the release of drugs responsive to the extra and intra pH of cancerous cells, which is very important for drug delivery systems. On the other hand, in 2015, Yao and co-workers [91] developed a kind of pH-responsive linker for the pores of mesoporous silica nanoparticles. They used PEGylated tetraphenylporphyrin zinc to act as the gate keeper for the controlled release of doxorubicin. Once the nano-platform reached the acidic extracellular pH of cancerous cells (pH = 6.8), the conjugated acid sensitive *cis*-aconitic anhydride bond between Zn and PEG was cleaved and caused DOX to be released.

Ruan and co-workers [112] synthesized pH-responsive polymeric micelles based on mPEG-PASP-benzaldehyde (PASP: polyaspartic acid), which is conjugated with DOX via a hydrazone linker. In other work, Wang and co-workers [123] prepared a new intelligent pH-sensitive nanoparticles based on UCNP (UFPLVs), which carried DOX by pH-sensitive hydrazone bonds on the surfaces of the nanocarrier. In 2018, a new type of pH-responsive COPs [220] was presented. In this study, acryloyl meso-tetra(*p*-hydroxyphenyl) porphine (acryloyl-THPP) reacted with 4,4′-trimethylene dipiperidine (TMPD) to form pH-responsive crosslinked biodegradable β-amino esters (BAEs) that can release DOX into the tumor microenvironment. 

#### 2.3.2. Enzyme Triggered

Different cancerous cells upregulate the expression of certain enzymes on their surface such as metalloproteinase and cathepsins. This phenomenon requires future studies to help researchers develop novel targeting agents according to those upregulated enzymes [261]. Along this line, Zhu and co-workers [27] used co-acervation technology to prepare a magnetic nanoparticle by gelatin and sodium alga acid. A citric acid/CuS@Fe_3_O_4_ nano-platform was prepared and DOX was loaded to be released by the enzymatic degradation of gelatin by the presence of gelatinase. It was observed that this nano-platform not only increased the accumulation of drugs in human breast cancer cells (MCF7), but also increased the level of ROS production. In 2019, Cui and co-workers [49] designed a SPNs based on a light-responsive backbone covered by PEG and conjugated with bromoisophosphoramide mustard intermediate (IPM-Br) via hypoxia-cleavable linkers [216]. Hypoxia in cancer cells precisely caused the fragmentation and release of IPM-Br catalyzed by nitroreductase, leading to cell death.

#### 2.3.3. Redox Triggered Agents

There are various reduction/oxidation (redox) reactions in cells, but cells continually regulate ROS levels to maintain natural physiological baseline levels. However, when cells turn cancerous, they alter their microenvironment, which can be exploited to develop drug delivery systems. There are special microenvironments in cancerous cells. In these cells, the level of glutathione and reactive oxygen species are higher than in healthy cells. Therefore, according to the level of glutathione in tumor cells, redox responsive drug delivery systems have been introduced to enhance the targeting ability of DDSs and decrease the side effects of therapeutic agents [262]. For example, glutathione responsive disulfide bonds were used to conjugate the anti-cancer agent camptothecin to iridium by Xiang and co-workers [31]. Liu and co-workers [99] designed poly(ethylene glycol)-based dendrimers (G3) that were conjugated to doxorubicin (anti-cancer drug) and porphyrin (photosensitizer) via disulfide bonds and Yi and co-workers [113] prepared polymeric prodrug poly(ethylene glycol)-b-poly(5-mthyl-5-propargyl-1,3-dioxan-2-one), which was grafted to paclitaxel by the disulfide bond. These nano-platforms could release the drug and photosensitizer by responding to the intracellular glutathione in cancer cells [263].

Platin prodrugs can also act as redox responsive materials. Their bond can be reduced to facilitate the anti-cancer drug platin to be released. For example, Lim and co-workers and Wang and co-workers used oxaliplatin prodrug cis-platinum to develop a redox responsive nano-platform. Both groups observed that platin could be released only by changing the levels of glutathione in tumor cells, which is very significant, in order to decrease the side effects of highly toxic platin-based drugs [264]. In other research, Gao and co-workers [108] developed polymeric micelles encapsulated with ZnPc as photosensitizers and DOX in which ZnPc was conjugated to the polymer by a redox-responsive disulfide linker. In 2019, Zhen and co-workers [115] reported a series of novel reduction-sensitive drug co-delivery systems based on fluorophores with strong NIR emission.

#### 2.3.4. Chemical and Biological Targeting Agents

Targeting ligands can be conjugated on the surface of nanocarriers to enhance accumulation and selectivity for certain cells or tissue types. Different materials such as hyaluronic acid, proteins/peptides, antibodies, carbohydrates/polysaccharides, aptamers, and folic acid have been tested for their ability to improve the selectivity of drug delivery systems [265]. For example, in 2019, Liang and co-workers [107] fabricated a nano-platform for synergistic effects based on GO to selectively deliver drugs into cancer cells that overexpress folate receptors.

CD44 usually binds with hyaluronic acid, which is one of the main materials of the extracellular matrix. Moreover, hyaluronic acid based materials can be degraded by hyaluronidase, which is abundant in tumor cells [266,267]. In 2019, Ren and co-workers [120] conjugated hyaluronic acid to chlorin-e6 and DOX via adipic di-hydrazide as a pH responsive linker. The result showed that the existence of hyaluronic acid could remarkably increase the cellular accumulation of DOX in A549 cells. In 2019, a new PDT-induced DDS was introduced by Zhu and co-workers [116], which was made of polyethylenimine-alkyl nitroimidazole (PA) and hyaluronic acid-chlorin-e6 to encapsulate TPZ. PA/hyaluronic acid (chlorin-e6)@TPZ NPs were capable of accumulating in the tumor site effectively due to hyaluronic acid-mediated cancer targeting. In another project, Zhou and co-workers [105] designed a smart nanosystem based on GO and hyaluronic acid surface modifications to improve and facilitate targeted delivery of CisPt and chlorin-e6 simultaneously.

Tumor homing peptides can be considered as other promising chemical targeting agents, for example, Lyp-1, which has the p32 protein (HABP1 or C1QR protein) as its receptor, can be conjugated to various drug delivery systems to increase their targeting abilities. Lyp-1 has nine amino acids and its receptors are overexpressed on the surface of cancer cell lines such as the 4T1, MDA-MB-435, and MCF-7 cell lines. Another peptide that can be considered as the targeting agent is the KE108 peptide, a synthetic nanopeptide that can efficiently target all five subtypes of somatostatin receptors overexpressed by neuroendocrine tumor cells. For example, Li and co-workers [110] conjugated Lyp-1 to cationic PCL grafted mPEG-PCL micelles, which were chosen as a carrier for docetaxel and IR820 (photosensitizer). They chose the 4T1 cell line, and it was observed that the presence of Lyp-1 could enhance the accumulation of micelles in the 4T1 cancer cell line, which was further proven by the receptor saturation technique. In another project, Shi and co-workers [114] developed a reactive oxygen species-responsive nanoparticle system to combine PDT and chemotherapy for oral tongue squamous cell carcinoma. A PEGlated prodrug of DOX via thioketal linkage and a peptide consist of cyclo-arginine-glycine-aspartic acid-d-phenylalanine-cysteine was synthesized and then used to prepare nanoparticles for the encapsulation of hematoporphyrin as the photosensitizer. In vivo experiments revealed that the nanocarriers had great targeting ability due to both thioketal and the conjugated peptide.

Antibody-targeted nanocarriers for cancer therapy have an exceptional role because of their specificity and vital advantages. Most monoclonal antibodies (mAbs) are used to target nanocarriers to cancer-specific antigens, deliver chemodrugs and photosensitizers in the form of antibody–drug conjugates, and recruit cytotoxic T cells to combat cancer cells [268]. For instance, Shiah and co-workers [269] reported the combination therapy of HPMA copolymer-bound DOX and M_chlorin-e6_ targeted with an OV-TL16 mAb in OVCAR-3 carcinoma xenografts. The OV-TL 16 antibody can identify the OA-3 antigen, which is expressed on OVCAR-3 cells and on most human ovarian carcinomas, and dramatically increased the accumulation of nanocarriers in tumors. In 2008, Hongrapipat and co-workers [227] confirmed increasing the biological activities of Fab′-targeted HPMA copolymers (Fab′ from OV-TL16 antibodies matching to CD47) loaded with the anticancer drug SOS thiophene and M_chlorin-e6_ over nontargeted conjugates. In 2018, Wang and co-workers [118] investigated unique multifunctional polysaccharide-based nanoparticles [270] with the anti-HER2 antibody as an active targeting agent [271] on the surface of the nanocarriers.

There are also other biological targeting agents. In 2016, He and co-workers [111] fabricated the design of NCP nanoparticles that carried oxaliplatin and the photosensitizer pyrolipid (NCP@pyrolipid) to significantly enhance antitumor immunity. NCP@pyrolipid combines two therapeutic modalities, chemotherapy and PDT, to elicit antitumor immunity [272], as evidenced by early calreticulin (CRT) exposure on the cell surface, antitumor vaccination, tumor-specific T-cell response, and an abscopal effect. The abscopal effect is usually described with ionizing radiation and refers to regression of the tumor outside the irradiated volume. Although the mechanism is unknown, it is thought to be immune modulated. More importantly, NCP@pyrolipid PDT treatment in combination with PD-L1 checkpoint blockade therapy led to the regression of the primary tumors that were locally treated with light irradiation and more interestingly, resulted in the regression of distant tumors in bilateral syngeneic mouse tumor models of CT26 and MC38. It was shown that this was achieved by generating a systemic tumor-specific T-cell response with the infiltration of CD8^+^ T cells and CD4^+^ T cells in distant tumors.

## 3. Conclusions

In the present review, various types of photosensitizers, their synthetic strategies, and the synergistic effect of PDT and chemotherapy were explored. This review split photosensitizers into two main categories that can be defined as with or without external carriers. In the first category, photosensitizers can act as either the drug or carrier, so there is no need for external carriers or drugs to achieve the synergistic effect of PDT and chemotherapy. In the second category, various external carriers such as transition metals, silica, graphene, liposomes, dendrimers, polymers, metal–organic frameworks, and different types of biological carriers are used to deliver photosensitizers and chemo–drugs to specific tumor sites. Furthermore, there are other strategies to increase the efficiency of treatment and decrease the side effects. For example, pH, redox, and enzyme triggered methods and/or surface modification by different chemicals have been used to increase the selectivity of photosensitizers and their carriers and potentially reduce or eliminate unwanted side effects. Although lots of photosensitizers have been proven to be efficient, the assessment of their cytotoxicity, biocompatibility, bio-distribution, and excretion are very important and are currently under investigation. Finding more convenient ways for treatment such as the oral administration of photosensitizers can further expand PDT to gain even more attraction. Finally, the mechanism of cellular uptake and the fate of different types of photosensitizers in the human body is key to their utilization. Appropriate strategies and model systems are required to obtain comprehensive knowledge about such complicated processes before using these materials in clinical trials. Eventually, we believe that with all of these therapeutic strategies and methods, the combination of PDT and chemotherapy has great potential and may soon move beyond model organisms.

## Figures and Tables

**Table 1:** Advantages and disadvantages of various nano-particles for cancer treatment.

Combination of PDT and Chemotherapy	Type of Carriers	Advantages	Disadvantages
Without external carriers	Photosensitizers as carriers	Faster synthesis process Simple compound Cost-effective	Possible toxicity Less-selectivity Fast elimination
	Photosensitizer-drug materials		
With external carriers	Transition metal based nano-platforms	Biocompatible Targeted drug carrier Selective Enhancing drug’s accumulation in the tumor site Tissue penetration Long time plasma half-life (stability)	Complex compound Longer synthesis process More cost Complex elimination routes
	Silica		
	Graphene		
	Liposomes		
	Dendrimers		
	Polymers		
	Metal–organic frameworks		
	Biological nanocarriers		
	Nano emulsions		

**Table 2:** Combination of photosensitizers and chemo-drugs without external carriers.

Reference	Photosensitizer (Carrier)	Drug
[27]	citric acid/CuS@Fe_3_O_4_	Doxorubicin
[28]	[(η6-p-cymene)Ru(2,3-bis(2-pyridyl)-benzoquinoxaline)(pyridine)]^2+^	Ru (II) segments
[29]	Porphyrin	Oxaliplatin-adamantane
[30]	Zinc phthalocyanine	Coumarin
[31]	Cyclometallated Ir(III) complex	Camptothecin
[32]	Cu_2−x_Se	Doxorubicin
[33]	NaYF4:Yb/Tm-TiO_2_	Doxorubicin
[34]	Silver nanoparticles	Doxorubicin
[35]	ZnO nanorods	Daunorubicin
[36]	MnO_2_-Pt@Au_25_	Platinum (IV) prodrugs
[37]	Zinc phthalocyanine	Ganetespib
[38]	Polyelectrolytes-NaYF4:Yb/Tm	Doxorubicin
[39]	AgFeO_2_	Quercetin
[40]	MnFe_2_O_4_	Curcumin
[40]	Cr_2_Fe_6_O_12_	Curcumin
[41]	Ti_3_C_2_ MXene	Doxorubicin
[42]	Ti_3_C_2_ MXene	Metformin
[43]	MoS_2_	Doxorubicin
[44]	Boron-dipyrromethene	Lenvatinib
[45]	porphyrin-containing Janus macromolecular brush	Doxorubicin
[46]	mPEG-Hydrazone-Br_2_-4,4-difluoro-4-bora-3a,4a-diaza-s-indacene	Doxorubicin
[47]	Ir(III)	Paclitaxel
[48]	Fe_3_O_4_@MnO_2_-Chlorin-e6	Traditional Chinese medicine

**Table 3:** Combination of photosensitizers and chemo-drugs with carriers.

Reference	Photosensitizer	Drug	Carrier
[75]	Photochlor	Prodrug banoxantrone	UiO-66-H/N_3_ (MOF)
[76]	Merocyanine 540	Doxorubicin	YbPO_4_:Er, Dy
[77]	Mitoxantrone	Mitoxantrone	PEGylated Hollow gold nanoparticles
[78]	Hematoporphyrin	Docetaxel	Gd-up conversion nanoparticles core/mesoporous silica shell
[79]	Chlorin core star shaped block copolymer	Camptothecin-11	Micelles
[80]	Rose Bengal	Platinum IV	NaGdF_4_:Yb/Nd@NaGdF_4_:Yb/Er@NaGdF_4_
[81]	Rose Bengal	AB_3_, a histone deacetylase inhibitor	NaYF4:Yb/Tm/Er
[82]	Merocyanine 540	Doxorubicin	NaYF4:Yb/Er
[83]	Chlorin-e6	Camptothecin	Up-conversion nanoparticles
[84]	Pyropheophorbide	Doxorubicin	Up-conversion nanoparticles
[85]	Chlorin-e6	*c*,*c*,*t*-[Diamine-dichlorodisuccinato-platinum(IV)]	[Mg_(1−x)_Al_x_(OH)_2_][A^n−^_x/n_]·zH_2_O
[86]	Zinc(II) phthalocyanine	Doxorubicin	Mesoporous silica nanoparticle
[87]	Chlorin-e6	Doxorubicin	Polyethylene glycol
[88]	Chlorin-e6	Doxorubicin	Mesoporous silica nanoparticle
[89]	Hematoporphyrin	Doxorubicin	Hollow Mesoporous Silica
[90]	Aluminum chloride phthalocyanine	Cisplatin	Mesoporous silica nanoparticle
[91]	PEGylated tetraphenylporphyrin zinc	Doxorubicin	Mesoporous silica nanoparticle
[92]	Chlorin-e6	Doxorubicin	Fe_3_O_4_@mSiO_2_(DOX)@ Human serum albumin
[93]	Fullerene (C60)	Doxorubicin	Mesoporous hollow silica
[94]	Chlorin-e6	Cisplatin	Mesoporous silica nanoparticle
[95]	2-[1-Hexyloxyethyl]-2-devinyl pyropheophorbide	Doxorubicin	Liposome
[96]	Chlorin-e6	Doxorubicin	Microbubble-lipid mixture
[97]	Indocyanine green-octadecylamine	Doxorubicin	Light sensitive liposome
[98]	IR780	Tirapazamine	Liposome
[99]	Porphyrin	Doxorubicin	Dendritic poly(ethylene glycol) (PEG-G3-OH) copolymer
[100]	5,10,15,20-Tetraphenylchlorin	Paclitaxel	Red blood cells membrane-camouflaged nanoparticles
[101]	Chlorin-e6	Doxorubicin	Hybrid protein oxygen carriers
[102]	Indocyanine green	Doxorubicin	Red blood cells containing oxyhemoglobin
[6]	Chloroaluminum phthalocyanine	Doxorubicin	Nano emulsions
[103]	CaFe_2_O_4_	Curcumin	Polyvinyl alcohol
[104]	Hypocrellin A	7-ethyl-10-hydroxycamptothecin	Graphene oxide
[105]	MnO_2_	Cis-Platine	Graphene oxide
[106]	4-Hydroxy coumarin	Camptothecin	Graphene oxide
[107]	Methylene blue	Doxorubicin	Graphene oxide
[108]	Zinc(II) phthalocyanine	Doxorubicin	Methoxypolyethylene glycol (mPEG) and poly(β-benzyl-l-aspartate)
[109]	Hematoporphyrin	Doxorubicin	Co-polymer containing arylboronic ester (BE)-modified with amphiphilic co-polymer (mPEG-PBAM).
[110]	NIR dye-IR820	Docetaxel	Methoxy-poly ethylene glycol-poly caprolactone
[111]	Pyrolipid	Oxaliplatin	1,2-distearoyl-sn-glycero-3-phosphocholine, cholesterol, 1,2-distearoyl-sn-glycero-3-phosphoethanolamine polyethylene glycol 2000
[112]	4,4-difluoro-4-bora-3a,4a-diaza-sindacene	Doxorubicin	mPEG-polyaspartic acid-benzaldehyde
[113]	Fluorogen photosensitizer	Paclitaxel	Poly(ethylene glycol)-b-poly(5-mthyl-5-propargyl-1,3-dioxan-2-one)
[114]	Hematoporphyrin	Doxorubicin	PEGylated (cyclo-arginine-glycine-aspartic acid-d-phenylalanine-cysteine) peptide
[115]	NIR fluorophore	Paclitaxel	Poly(ethylene glycol)-b-poly(5-mthyl-5-propargyl-1,3-dioxan-2-one)
[116]	Hyaluronic Acid-chlorin-e6	Tirapazamine	Self-assembling amphiphilic polyethylenimine-alkyl nitroimidazole
[117]	Zn	Docetaxel	Co-polymers poly(ethylene oxide)-poly(ε-caprolactone)-poly(ethylene oxide)
[118]	5-aminolevulinic acid	Doxorubicin	Hydroxyethyl chitosan and aldehyde-functionalized hyaluronic acid
[119]	Mesotetra(p-hydroxyphenyl) porphine	Cis-platinum	Mesotetra(p-hydroxyphenyl)-Pt-PEG (covalent-organic polymers)
[120]	Chlorin-e6	Doxorubicin	Hyaluronic acid-chlorin-e6
[121]	C60	Doxorubicin	C60–PEI–DOX
[122]	Chlorin-e6	Doxorubicin	(ε-caprolactone-co-lactide)-b-poly (ethylene glycol)-b-poly (ε-caprolactone-colactide)
[123]	Merocyanine 540	Doxorubicin	UCNP-loaded (NaYF_4_:Yb, Er) folate-conjugated polymeric (dextran)
[124]	Pyropheophorbide-a	gemcitabine	Human serum albumin
[125]	Zinc phthalocyanine	Doxorubicin	[methoxy-poly(ethylene glycol)-poly(2-(N,N-diethylamino)ethyl methacrylate)-poly(ε-caprolactone)]4-zinc β-tetra-(4-carboxyl benzyloxyl)phthalocyani
[126]	Indocyanine green	Doxorubicin	Nano-scaled red blood cells
[127]	Purpurin 18	Doxorubicin	mPEG-Cyclodextrin-Polyhydroxybutyrate
[128]	Gold nanoclusters	Doxorubicin	(ZIF-8) metal–organic framework
[129]	protoporphyrin IX	Doxorubicin	(ZIF-8) metal–organic framework
[130]	Chlorin-e6	Tirapazamine	(polyethylene glycol)-*Azo-benzene*-poly (d, l-lactide-*co*-glycolide)
[131]	Chlorin-e6	Gambogic acid	Hyaluronic acid-nitroimidazole (HA-NI) as shells, MnO_2_ NPs functionalized poly (l-glutamic acid) derivatives (γ-PFGA) as cores
[132]	Si photosensitizer	Doxorubicin	Mesoporous silica nanoparticle
[133]	Chlorin-e6	Doxorubicin	Polyoligo (ethylene glycol) methacrylate-block-poly(ε-caprolactone)-azobenzene-poly(ε-caprolactone)-block-poly oligo (ethylene glycol)
[134]	Pyropheophorbide	paclitaxel	Poly [oligo (ethylene glycol) methyl ether methacrylate]
[135]	Pheophorbide a	Tirapazamine	Self-assembled gelatin nanoparticles
[136]	Chlorin-e6	Doxorubicin	Poly(phosphorylcholine)
[137]	Chlorin-e6	Oridonin	Side-chain selenium-grafted polymers
[138]	porphyrin	Doxorubicin	Tetra-β-cyclodextrin
[139]	Chlorin-e6	Gemcitabine	Polymeric micelles
[140]	Chlorin-e6	Gemcitabine	Multifunctional polymeric prodrug micelles
[141]	Chlorin-e6	Paclitaxel	Liposomes
[142]	Chlorin-e6	Docetaxel	Hyaluronic acid
[143]	pyropheophorbide-a	camptothecin	mPEG with thioketal linker
[144]	Chlorin-e6	Doxorubicin	Block copolymers polystyrene-b-poly (acrylic acid) and oil-soluble
[145]	Chlorin-e6	Perfluorohexanoate-modified cisplatin	Poly(ethylene glycol)-lysine-block-poly(L-glutamate)-imidazole
[146]	protoporphyrin IX	Tegafur (prodrug of 5-fluorouracil)	Heterodimers hydrogel
[147]	zinc phthalocyanine	Tirapazamine	Hyaluronic acid
[148]	Chlorin-e6	cisplatin	Dual-effect liposome
[149]	Porphyrin	Paclitaxel	Porphyrin-lipid shelled nano-emulsion
[150]	5-aminolevulinic acid to produce protoporphyrin IX	Doxorubicin	Nanogel

## Data Availability

Not applicable.

## Abbreviations

**Abbreviations**	**Full Names**
PDT	Photodynamic therapy
PS	Photosensitizer
ROS	Reactive oxygen species
^1^O_2_	Singlet oxygen
SPNpd	Semiconducting polymer nano-prodrug
NIR	Near-infrared
2D	Two dimensional
SP	Soybean phospholipids
PAI	Photoacoustic imaging
IONP	Iron oxide nanoparticles
DMSO	Dimethyl sulfoxide
TBAOH	Tetrabutylammonium hydroxide
TMAOH	Tetramethylammonium hydroxide
LSPR	Localized surface plasmon resonance
CP	Compound polysaccharide
Met	Metformin
DOX	Doxorubicin
PEG	Polyethylene glycol
PNBMA	Poly (4,5-dimethoxy-2-nitrobenzyl methacrylate)
HSA	Human serum albumin
GO	Graphene oxide
rGO	Reduced graphene oxide
CVD	Chemical vapor deposition
NMP	*N*-methyl-pyrrolidone
SDBS	Sodium dodecylbenzene sulfonate
GSH	Glutathione
CPT	Camptothecin
MB	Methylene blue
FA	Folic acid
PAMAM	Polyamidoamine
LED	Light emitting diode
SPN	Semiconducting polymer nanoparticles
ZnPc	Zinc(II) phthalocyanine
Hp	hematoporphyrin
PASP	Polyaspartic acid
TPZ	Tirapazamine
DTX	Docetaxel
LBL	Layer-by-layer
Pt	Cis-platinum
PTX	Paclitaxel
Gem	Gemcitabine
Rf	Riboflavin
MOF	Metal–organic framework
HPPH	Photochlor
MDR	Multidrug resistance
P-gp	P-glycoprotein
mAbs	Monoclonal antibodies
